# Author Correction: Non-solvent post-modifications with volatile reagents for remarkably porous ketone functionalized polymers of intrinsic microporosity

**DOI:** 10.1038/s41467-023-40301-1

**Published:** 2023-07-31

**Authors:** Sirinapa Wongwilawan, Thien S. Nguyen, Thi Phuong Nga Nguyen, Abdulhadi Alhaji, Wonki Lim, Yeongran Hong, Jin Su Park, Mert Atilhan, Bumjoon J. Kim, Mohamed Eddaoudi, Cafer T. Yavuz

**Affiliations:** 1 Department of Chemical and Biomolecular Engineering, Korea Advanced Institute of Science and Technology (KAIST), 291 Daehak-ro, Yuseong-gu, Daejeon, 34141 Republic of Korea; 2 PTT Global Chemical Public Company Limited, Bangkok, 10900 Thailand; 3 Oxide & Organic Nanomaterials for Energy & Environment Laboratory, Physical Science & Engineering (PSE), King Abdullah University of Science and Technology (KAUST), Thuwal, 23955 Saudi Arabia; 4 Advanced Membranes & Porous Materials Center, PSE, KAUST, Thuwal, 23955 Saudi Arabia; 5 KAUST Catalysis Center, PSE, KAUST, Thuwal, 23955 Saudi Arabia; 6 Department of Chemical and Paper Engineering, Western Michigan University, Kalamazoo, MI 49008-5462 USA

**Keywords:** Polymers, Organic molecules in materials science, Polymers

Correction to: *Nature Communications* 10.1038/s41467-023-37743-y, published online 13 April 2023

The original version of this Article contained an error in Fig. 2 and supplementary Figures 1, 2, 3 and 48, in which the structure of PIM-1 is displayed wrongly. The correct version of Fig. 2 and Supplementary Figures 1, 2, 3 and 48 are:


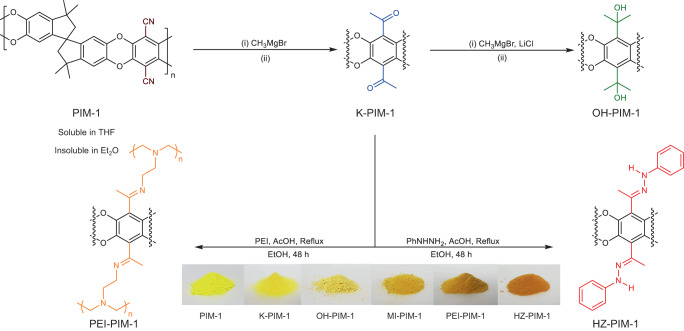


**Fig. 2: The quantitative post-modification of PIM-1 through conventional and non-solvent methods with a volatile Grignard reagent, methylmagnesium bromide (CH**_**3**_**MgBr)**. Ketone-PIM−1 (K-PIM-1) was further reacted by the same reagent to make Alcohol-PIM−1 (OH-PIM-1) with remarkably high retention of porosity, considering the two-step reaction. Solvent: (i) CH_3_MgBr in tetrahydrofuran (THF), THF solvent at 0 °C → RT for 24 h (ii) 0.5 M hydrochloric acid (HCl) in methanol (MeOH), H_2_O at 60 °C for 4 h. Non-solvent: (i) CH_3_MgBr in diethyl ether (Et_2_O) at 0 °C → RT for 24 h (ii) 0.5 M HCl in MeOH, H_2_O at 60 °C for 4 h. K-PIM−1 was further functionalized by phenylhydrazine (PhNHNH_2_) and polyethylenimine (PEI) to show versatility in the reactive portfolio and to create CO_2_ adsorbents.


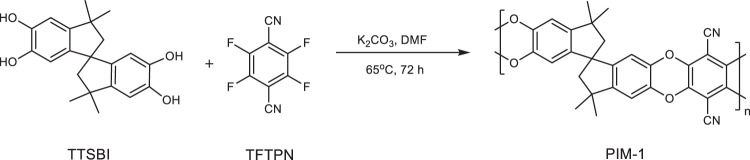


**Supplementary Figure 1:** Synthesis of PIM-1 via low temperature approach.


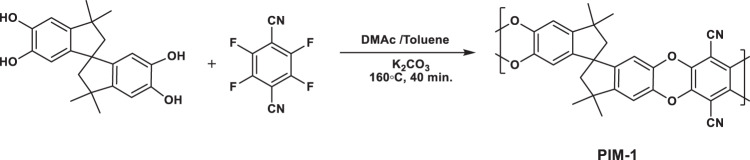


**Supplementary Figure 2:** Synthesis of PIM-1 via high temperature approach.


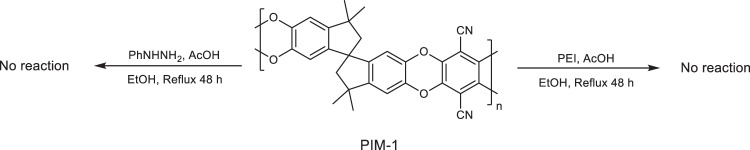


**Supplementary Figure 3:** Control reaction using PIM-1 as a starting material to react with phenylhydrazine (PhNHNH_2_) and polyethylenimine (PEI), refluxed in the presence of ethanol solvent (EtOH) under mild acidic condition.


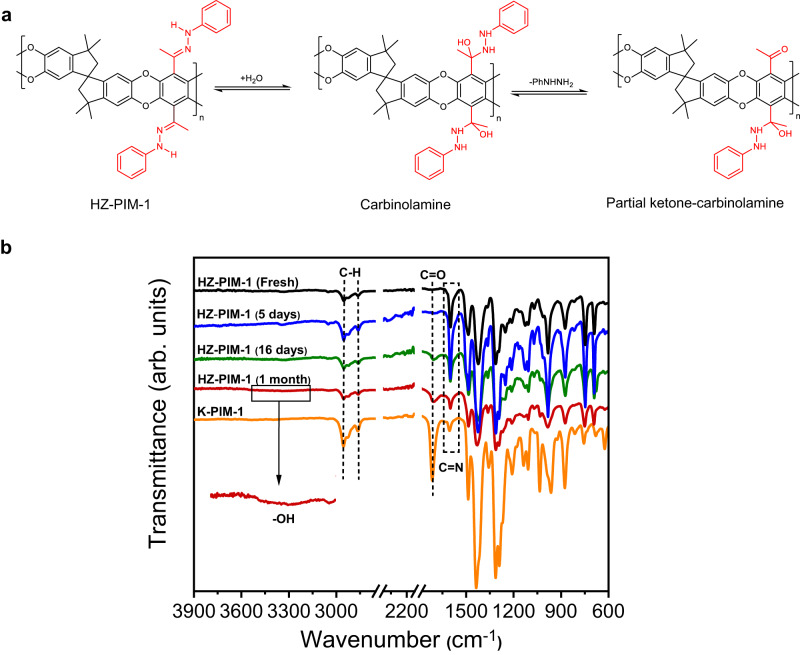


**Supplementary Figure 48: a** Mechanism and **b** FT-IR spectra revealed the reversible reaction of HZ-PIM-1.

which replace the previous incorrect versions:


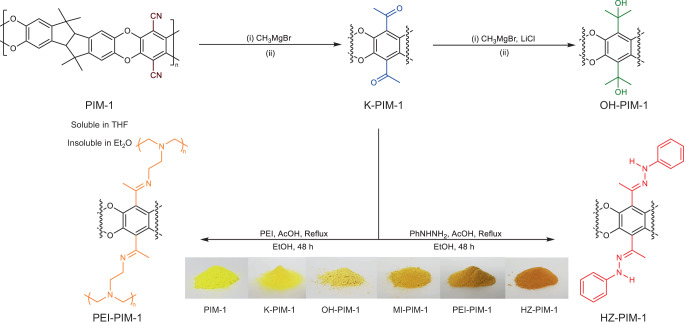


**Fig. 2:** **The quantitative post-modification of PIM-1 through conventional and non-solvent methods with a volatile Grignard reagent, methylmagnesium bromide (CH**_**3**_**MgBr)**. Ketone-PIM−1 (K-PIM-1) was further reacted by the same reagent to make Alcohol-PIM−1 (OH-PIM-1) with remarkably high retention of porosity, considering the two-step reaction. Solvent: (i) CH_3_MgBr in tetrahydrofuran (THF), THF solvent at 0 °C → RT for 24 h (ii) 0.5 M hydrochloric acid (HCl) in methanol (MeOH), H_2_O at 60 °C for 4 h. Non-solvent: (i) CH_3_MgBr in diethyl ether (Et_2_O) at 0 °C → RT for 24 h (ii) 0.5 M HCl in MeOH, H_2_O at 60 °C for 4 h. K-PIM−1 was further functionalized by phenylhydrazine (PhNHNH_2_) and polyethylenimine (PEI) to show versatility in the reactive portfolio and to create CO_2_ adsorbents.


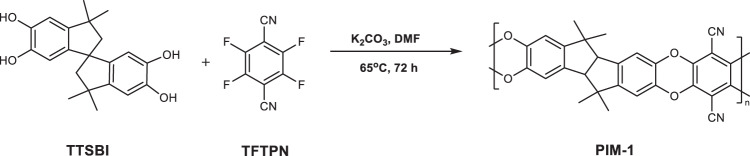


**Supplementary Figure 1:** Synthesis of PIM-1 via low temperature approach.


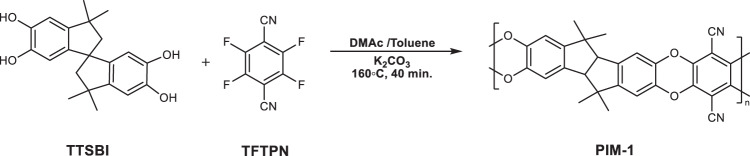


**Supplementary Figure 2:** Synthesis of PIM-1 via high temperature approach.


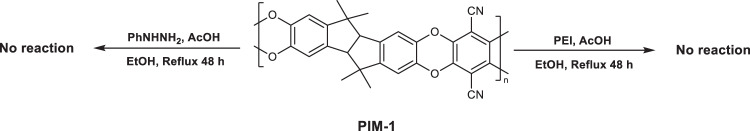


**Supplementary Figure 3:** Control reaction using PIM-1 as a starting material to react with phenylhydrazine (PhNHNH_2_) and polyethylenimine (PEI), refluxed in the presence of ethanol solvent (EtOH) under mild acidic condition.


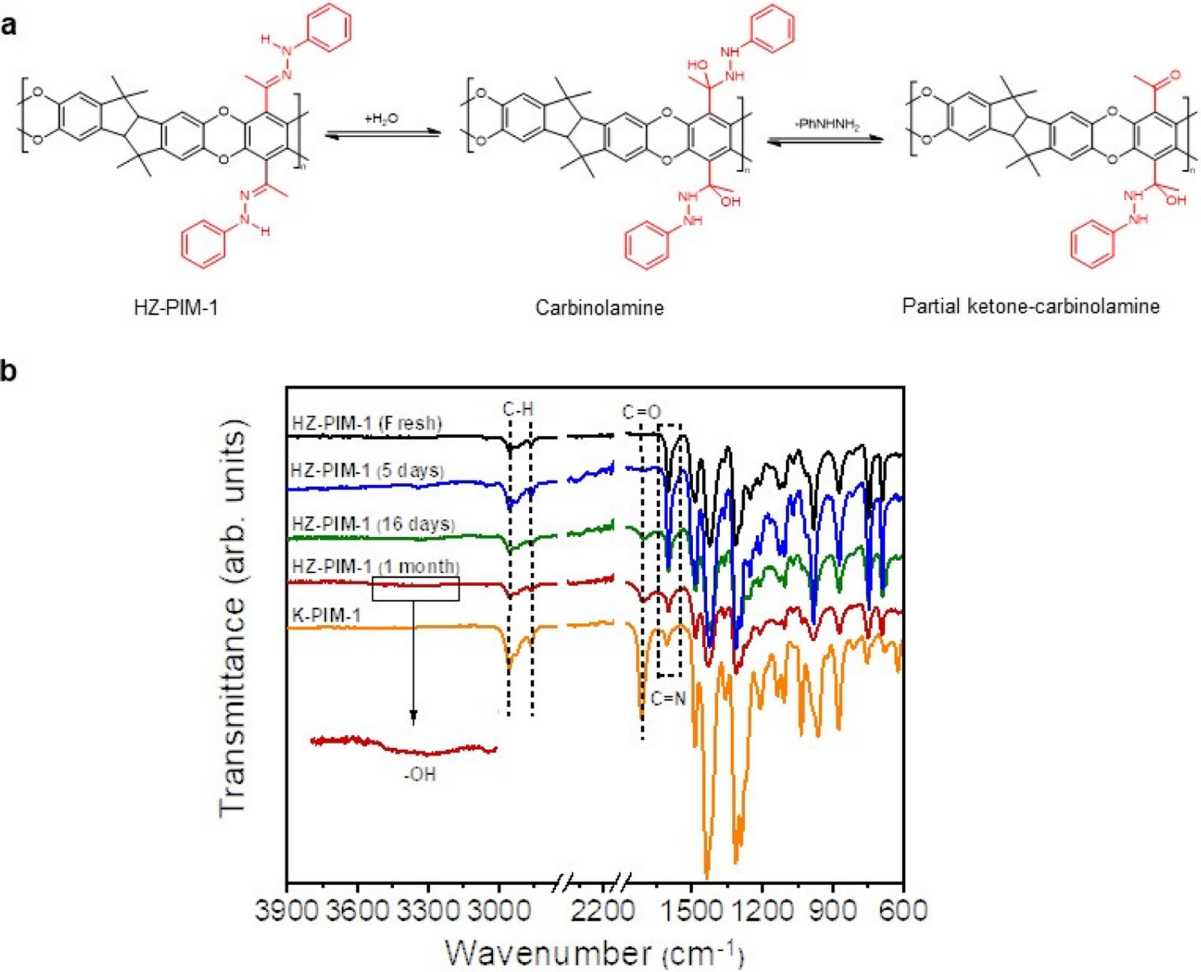


**Supplementary Figure 48:** **a** Mechanism and **b** FT-IR spectra revealed the reversible reaction of HZ-PIM-1.

This has been corrected in both the PDF and HTML versions of the Article.

## Supplementary information

Updated Supplementary Information

