# Supplementary material for: Non-solvent post-modifications with volatile reagents for remarkably porous ketone functionalized polymers of intrinsic microporosity
Source: Nat Commun. 2023 Apr 13;14:2096. doi: 10.1038/s41467-023-37743-y (PMC10102017; doi:10.1038/s41467-023-37743-y)
Supplement: Supplementary file 1 — Supplementary Information [file 41467_2023_37743_MOESM1_ESM.pdf]

Supplementary Information

Non-solvent post-modifications with volatile reagents for remarkably porous ketone functionalized polymers of intrinsic microporosity

Sirinapa Wongwilawan[1, 2], Thien S. Nguyen[3, 4, 5], Thi Phuong Nga Nguyen[3], Abdulhadi Alhaji[4], Wonki Lim[1], Yeongran Hong[1], Jin Su Park[1], Mert Atilhan[6], Bumjoon J. Kim[1], Mohamed Eddaoudi[4], Cafer T. Yavuz[1, 3, 4, 5*]

[1]*Department of Chemical and Biomolecular Engineering, Korea Advanced Institute of Science and Technology (KAIST), 291 Daehak-ro, Yuseong-gu, Daejeon, 34141, Republic of Korea*

[2] *PTT Global Chemical Public Company Limited, Bangkok 10900, Thailand*

[3] *Oxide & Organic Nanomaterials for Energy & Environment Laboratory, Physical Science & Engineering (PSE), King Abdullah University of Science and Technology (KAUST), Thuwal 23955, Saudi Arabia*

[4] *Advanced Membranes & Porous Materials Center, PSE, KAUST, Thuwal 23955, Saudi Arabia*

[5] *KAUST Catalysis Center, PSE, KAUST, Thuwal 23955, Saudi Arabia*

[6] *Department of Chemical and Paper Engineering, Western Michigan University, Kalamazoo, MI 49008-5462, USA*

*Email: cafer.yavuz@kaust.edu.sa (Cafer T. Yavuz)

Contents

1. Synthesis of PIM-1 3

    1.1 Low temperature method (LT)[1, 2] 3

    1.2 High temperature method (HT)[3, 4] 4

2. Proof of concept 5

3. Processability 6

4. Calculation of Surface Areas[5, 6] 7

5. CO_2 Breakthrough Experiment 8

    5.1 Methodology 8

    5.2 Derivation of adsorption capacity 9

    5.3 Calculation from experimental data: 10

6. Supplementary Table and Figure 11

7. Supplementary References 53

1. Synthesis of PIM-1

1.1 Low temperature method (LT)[1, 2]

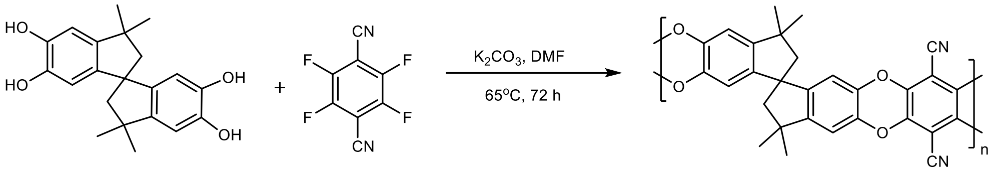

TTSBI TFTPN PIM-1

Supplementary Figure 1: Synthesis of PIM-1 via low temperature approach.

PIM-1 was synthesized via the low temperature (LT) method reported by Budd et al. and Patel et al. with a slight modification. 5,5’,6,6'-tetrahydroxy-3,3,3’,3’-tetramethyl-1,1'-spirobisindane (TTSBI) (3.4 g, 10 mmol) and tetrafluoroterephthalonitrile (TFTPN) (2 g, 10 mmol) were added to a desired reaction solvent (70 mL) of anhydrous *N,N*-dimethyl formamide in a dry three-neck round-bottom flask under inert atmosphere of argon. The reaction mixture was stirred at 65 °C until a clear solution was observed. Fine potassium carbonate (4.15 g) was gradually added to the system, and then the reaction mixture was stirred for 72 hours. At the end of the reaction, a highly viscous solution was cooled down and poured into water (650 mL). The polymer solid was obtained by filtration. The purification was performed by dissolving the material in chloroform (160 mL) and precipitating from methanol (500 mL) twice. The precipitated PIM-1 was then vacuum filtered and washed with 1,4-dioxane (100 mL), acetone (100 mL), water (100 mL), and an excess amount of methanol. The luminous yellow product was dried in a vacuum oven at 120 °C overnight (Yield: 78.2%, 3.60 g). PIM-1 powder was stored in a tightly sealed container to prevent moisture contact.

1.2 High temperature method (HT)[3, 4]

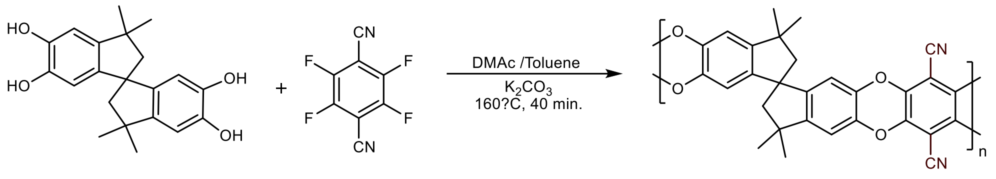

Supplementary Figure 2: Synthesis of PIM-1 via high temperature approach.

The high temperature method of PIM-1 was synthesized following the previous report of Huajie Y. et al. The starting materials; 5,5',6,6'-tetrahydroxy-3,3,3',3'-tetramethyl-1,1'-spirobisindane (TTSBI) (4.26 g, 12.5 mmol), tetrafluoroterephthalonitrile (TFTPN) (2.50 g, 12.5 mmol), fine anhydrous potassium carbonate (5.18 g, 37.5 mmol), dimethylacetamide DMAc (25 mL), and toluene (12.5 mL) were placed to a dry three-neck round bottom flask equipped with a Dean-Stark trap and condenser under an atmosphere of argon gas. The reaction mixture was left to stir at ambient temperature until TTSBI and TFTPN were completely dissolved. Then the solution mixture was refluxed at 160°C in an oil-bath with vigorous stirring for 40 min. Subsequently, the viscous solution was poured into methanol to acquire a yellow polymer solid. After filtration, the sample was dissolved in chloroform (100 mL) and re-precipitated from methanol. The final product was further stirred in 1,4-dioxane (50 mL) for 30 min to remove low molecular weight oligomers and cyclic products. The sample was collected and re-washed with acetone. To eliminate K_2CO_3 residue, the PIM-1 powder was refluxed overnight in deionized water, stirred in methanol for 20 min, and then dried at 120 °C in a vacuum oven overnight. The fluorescent yellow polymer (PIM-1) product yielded 4.31 g (91.5%).

2. Proof of concept

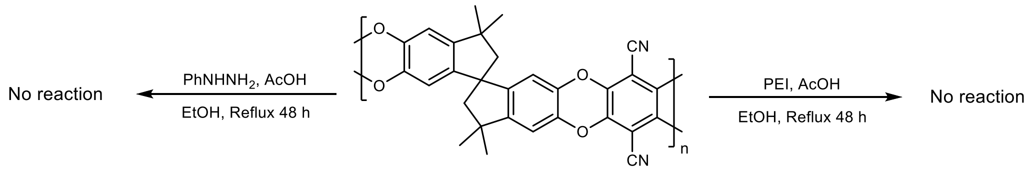

PIM-1

Supplementary Figure 3: Control reaction using PIM-1 as a starting material to react with phenylhydrazine ($PhNHNH_2$) and polyethylenimine (PEI), refluxed in the presence of ethanol solvent (EtOH) under mild acidic condition.

To confirm the indispensability of the ketone functional group for chemically anchoring with amine, the identical reaction conditions of amine functionalization of K-PIM-1 was performed by replacing K-PIM-1 with PIM-1. Phenylhydrazine ($PhNHNH_2$) and polyethylenimine (PEI) were used as reagents. In a dry three-neck round bottom flask equipped with a reflux condenser, PIM-1 (0.69 g, 1.5 mmol) was suspended in ethanol (EtOH) (15 mL). The mild acidic condition was induced by adding glacial acetic acid (AcOH) (0.45 mL) and heating up to 80 °C under argon atmosphere. After the temperature reached the setpoint, different amine solutions (0.76 mL of phenylhydrazine reagent or 5.7 mL of polyethylenimine) were added to the solution depending on the targeted final product, and the reaction was refluxed for 48 h. Then the reactions were cooled down to room temperature. The final products were filtered and gradually washed with ethanol (4x50 mL). Additionally, the samples were stirred in ethanol (100 mL) for 20 min before being filtered and dried in the vacuum oven at 105 °C for 12 h. The final products remained fluorescent yellow (Yield: 0.55 g and 0.62 g for the functionalization of PIM-1 with $PhNHNH_2$ and PEI, respectively).

3. Processability

a) PIM-1 film preparation: PIM-1 powder (0.5 g) was dissolved in chloroform (20 mL) and stirred overnight. After that, the homogenized polymer solution was poured into a flat-bottomed glass petri dish. The solvent was slowly evaporated in the glass cover for 3 days and further dried at 60 °C in a vacuum oven for 24 h.

b) K-PIM-1 film preparation: The dried PIM-1 film was cut into a small piece and transferred to the round bottom. Methylmagnesium bromide in diethyl ether (30 mL, non-solvent for PIM-1) was added to the system. The solution was stirred overnight. Then the workup process was performed by placing the round bottom in an ice bath for 15 min. Then 0.5 M HCl in methanol solution (85 mL) was slowly added to the solution. Afterwards 0.5 M HCl in an aqueous solution was slowly added to adjust pH in the range of 4-5. To complete conversion, the solution was additionally stirred at 60 °C for 4 h. The sample was filtered off and washed with an excess amount of water following by methanol. The film was allowed to dry in air overnight, and kept in a vacuum oven at 60 °C for 24 h.

4. Calculation of Surface Areas[5, 6]

To calculate the surface areas from N_2 adsorption isotherms at 77 K, we applied following criteria:

1) The linear fit should span at least 5 points.

2) The R^2 should be greater or equal to 0.995.

3) Over the entire fitting range $Q(1-P/P_0)$ must continuously increase with P/P_0

4) The value of C intercept obtained by linear regression must be positive in the plot of $1/[Q(P_0/P-1)]$ against P/P_0

The amount of gas molecules adsorbed in the initial monolayer is

$$Q_{\mathrm{m}} = \frac{1}{\mathrm{Slope+Intercept}} \quad (1)$$

The specific surface area was calculated as follows:

$$SA_{\mathrm{BET}} = Q_{\mathrm{m}}\left(\frac{\mathrm{cm}^3}{\mathrm{g}}\right) \times \frac{1\ (\mathrm{mol})}{22400\ (\mathrm{cm}^3)} \times 16.2\ (\text{Å}^2) \times N_{\mathrm{A}}\ (\mathrm{mol}^{-1}) \times 10^{-20}\left(\frac{\mathrm{m}^2}{\text{Å}^2}\right) \quad (2)$$

Where N_{A} is Avogadro's constant, and 16.2 Å^2 is the cross-sectional area of a N_2 molecule.

5. CO_2 Breakthrough Experiment

5.1 Methodology

Well-ground polymer was filled into a stainless-steel column and activated at 120 °C for 5 hours prior to breakthrough adsorption test. Before each experiment, helium reference gas was flushed through the column and the gas flow was then switched to the desired gas mixture at the flow rate of 5 mL min^{-1} (80.75% N_2, 14.25% CO_2, 5% He). The test was carried out at 1 bar pressure and 25 °C. Outcoming gases were analyzed by a gas analysis system (QGA, Hiden analytical, United Kingdom). The blank tests were conducted with empty column to suppress effect of system configuration on adsorption calculation.

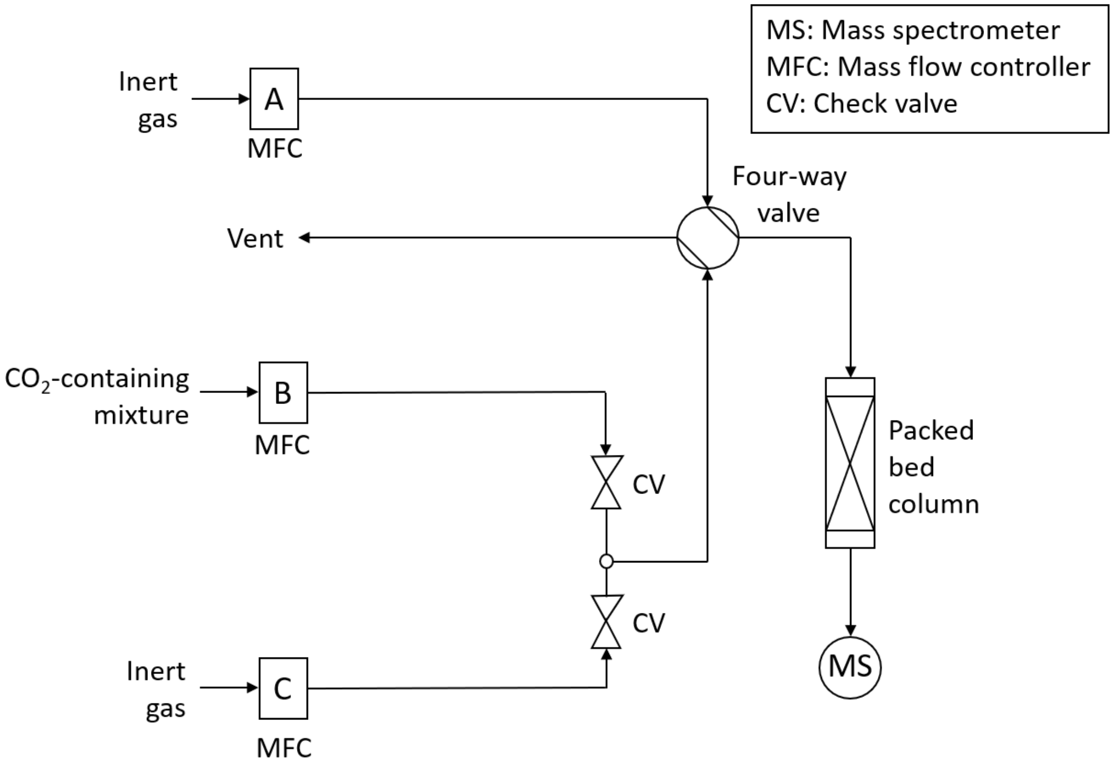

Supplementary Figure 4: Schematic diagram of the column breakthrough experiment.

5.2 Derivation of adsorption capacity

Adsorption capacity is calculated as the amount of uptake adsorbate per unit mass of the adsorbent.

$$q_{\mathrm{t}} = \frac{m_{\mathrm{ad}}}{m} \tag{1}$$

While,

$$m_{\mathrm{ad}} = C_{\mathrm{ad}}V \tag{2}$$

$$V = Ft \tag{3}$$

$$C_{\mathrm{ad}} = C_o - C_t \tag{4}$$

Substitute (2), (3) and (4) into (1).

$$q_{\mathrm{t}} = \frac{F}{m}(C_{\mathrm{o}} - C_{\mathrm{t}})t \tag{5}$$

Then total adsorbate will be calculated by Eq. (6)

$$q = \frac{F}{m}\int_0^{t_s}(C_o - C_t)dt$$

or

$$q = \frac{FC_o}{m}\int_0^{t_s}\left(1 - \frac{C_t}{C_o}\right)dt \tag{6}$$

Where,

q	: Adsorption capacity (mmol g^{-1})
q_t	: Adsorption capacity at time t (mmol g^{-1})
m_{ad}	: Mass of adsorbate (g)
m	: Mass of adsorbent (g)
C_{ad}	: Concentration of adsorbate taken by absorbent (g)
V	: Volume of adsorbate (L)
F	: Flow rate of adsorbate (L min^{-1})
t	: Adsorption time (min)

C_o : Initial concentration of adsorbate (mmol L^{-1})

C_t : Concentration of adsorbate come out from fixed bed (mg L^{-1})

t_s : Saturated time (min)

5.3 Calculation from experimental data:

F = 5 mL min^{-1} CO_2 = 0.005 L min^{-1}

$C_o = \frac{1}{24.76\text{L}} \times 14.25\%$ = 5.755 mmol L^{-1}

(At 1 bar, 25 ^oC, 1 mole of ideal gas is 24.76 L)

m = 181 mg of PIM-1 and 236 mg PEI-PIM-1

$\int_0^{t_s}\left(1-\frac{C_t}{C_o}\right)dt$ is the subtracted area between the plot $\frac{C}{C_o}=1$ and $\frac{C}{C_o}=\frac{C_t}{C_o}$ versus time t

For blank test, $\int_0^{t_s}\left(1-\frac{C_t}{C_o}\right)dt = 1.253$

For PIM-1, $\int_0^{t_s}\left(1-\frac{C_t}{C_o}\right)dt = 3.228$

Then adsorption capacity (q) of PIM-1 is 0.31 mmol g^{-1}.

For PEI-PIM-1 $\int_0^{t_s}\left(1-\frac{C_t}{C_o}\right)dt = 6.011$

Then adsorption capacity (q) of PEI-PIM-1 is 0.58 mmol g^{-1}.

6. Supplementary Table and Figure

Supplementary Table 1: Comparison of molecular weight and film formation of PIM-1 derived from different synthesis methods: low temperature method (LT) and high temperature method (HT).

PIM-1	GPC result			Film formation
	M_n	M_w	PDI	
Low temperature method (LT)	42913	76648	1.79	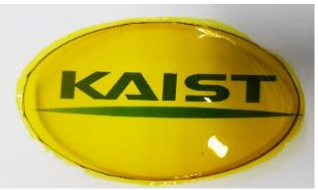
	50164	89015	1.77	
High temperature method (HT)	25005	37816	1.51	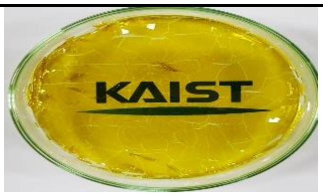
	14609	23621	1.61	

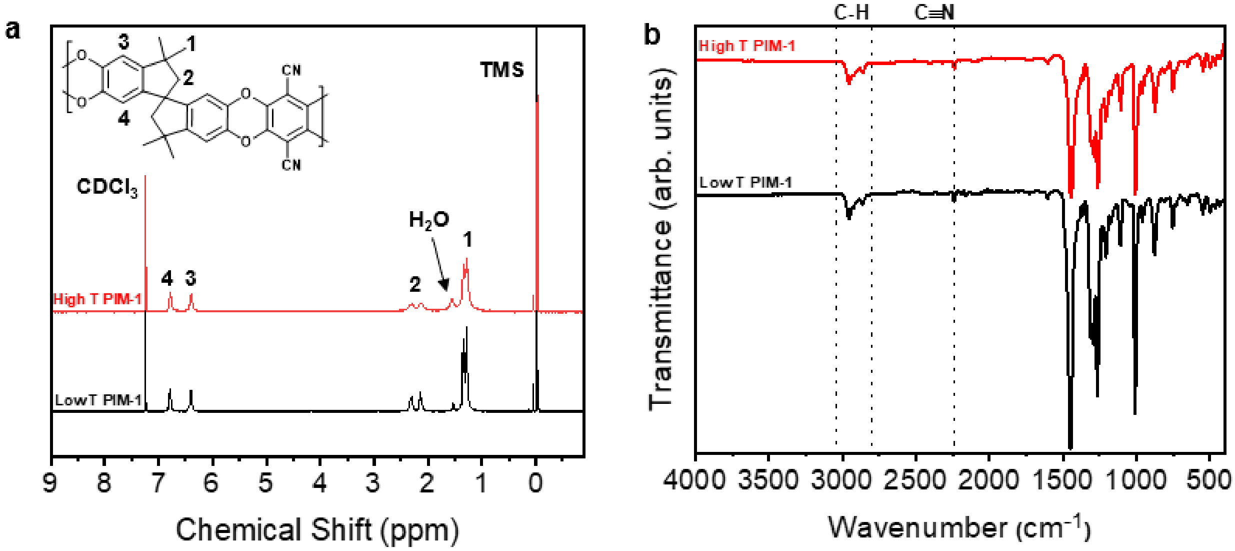

Supplementary Figure 5: a ^{1}H NMR and **b** FT-IR spectra comparison of high temperature and low temperature methods of PIM-1.

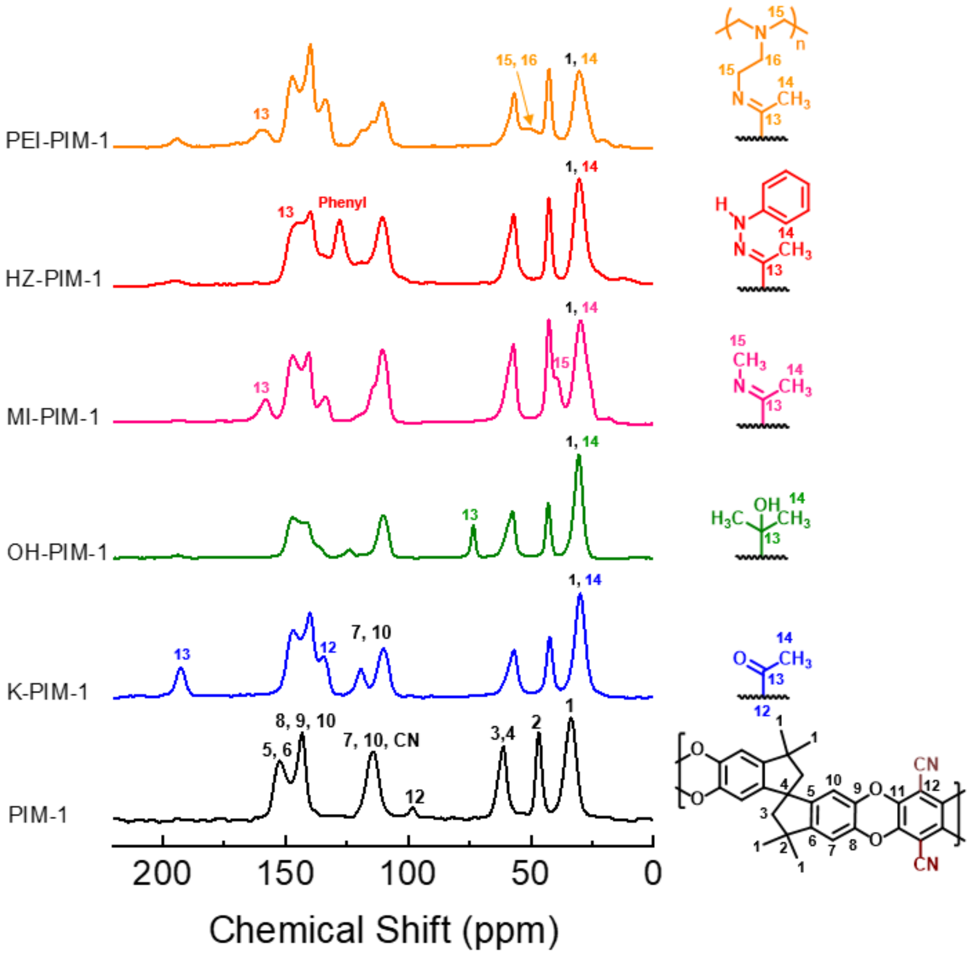

Supplementary Figure 6: Solid-state ^{13}C CP-MAS NMR spectrum of PIM-1 and derivatives.

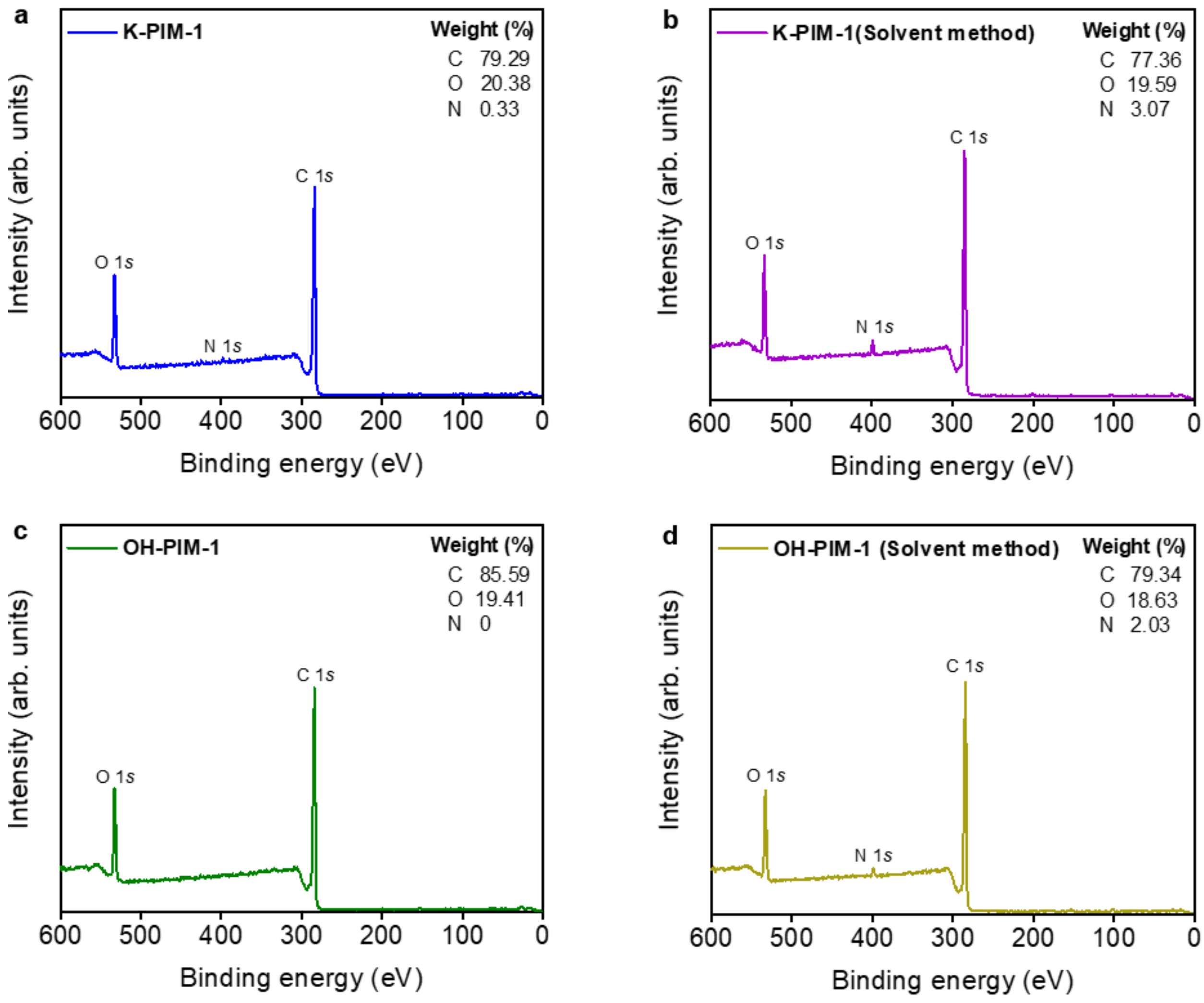

Supplementary Figure 7: XPS survey scan of **a** K-PIM-1 derived from non-solvent method, **b** K-PIM-1 (solvent method) derived from solvent method, **c** OH-PIM-1 derived from non-solvent method, and **d** OH-PIM-1 (solvent method) derived from solvent method.

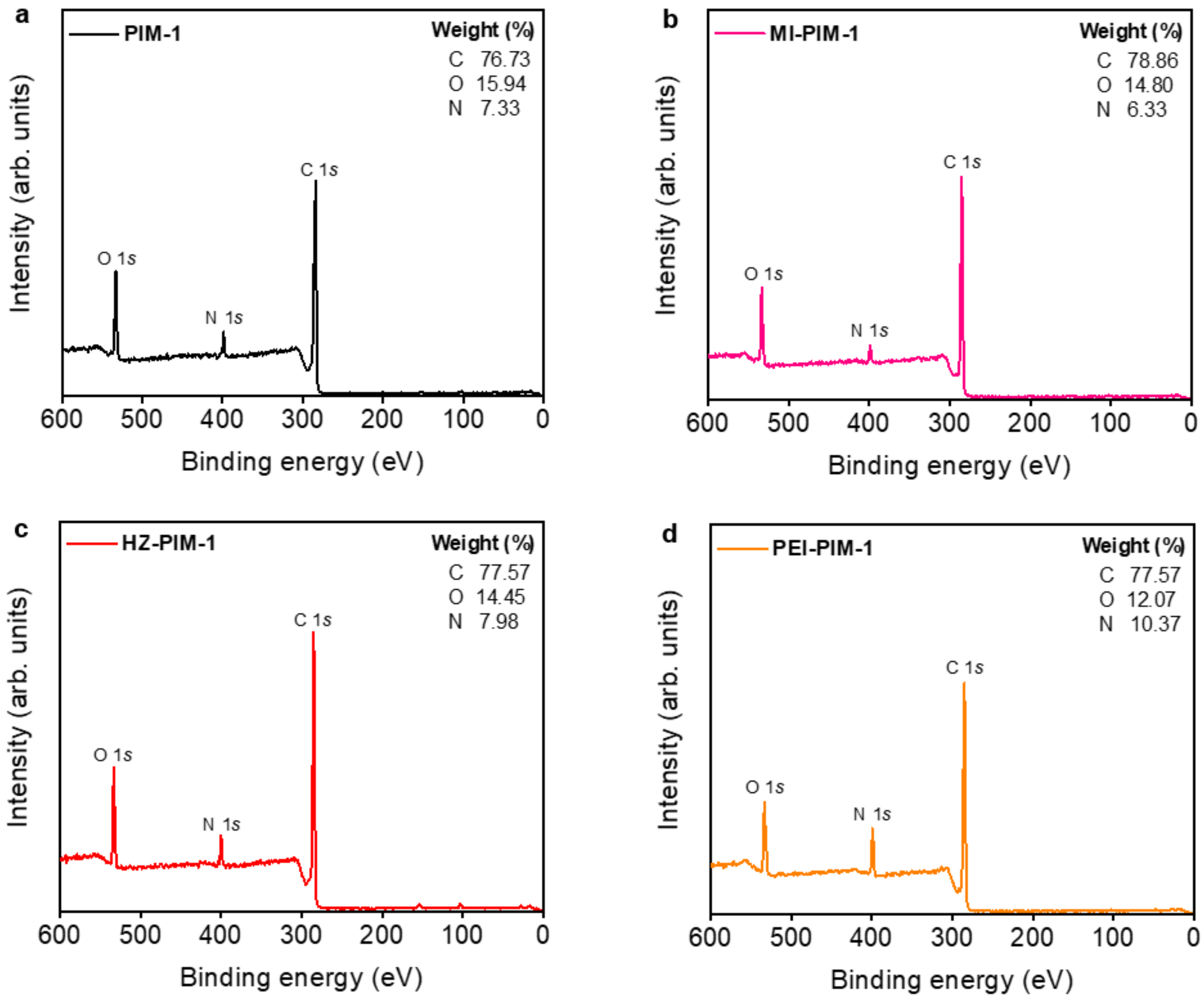

Supplementary Figure 8: XPS survey scan of **a** PIM-1, **b** MI-PIM-1, **c** HZ-PIM-1, and **d** PEI-PIM-1.

Supplementary Table 2: Reproducibility of K-PIM-1 synthesized via the non-solvent method.

No.	Elemental content				%Conversion	Surface area (m^2 g^{-1})
	%C	%H	%N	%O		
Theo.	75.29	5.30	0	19.41		
Batch 1	74.30±0.31	5.37±0.05	0.45±0.01	17.90±0.40	92.9	651
Batch 2	74.43±0.00	5.20±0.03	0.59±0.00	17.85±0.44	90.6	705
Batch 3	74.09±0.02	5.19±0.01	0.46±0.02	18.09±0.06	92.7	703
Batch 4	74.35±0.07	5.22±0.00	0.42±0.00	18.27±0.02	93.3	704
Batch 5	73.86±0.07	5.23±0.02	0.41±0.01	18.09±0.10	93.5	692
Batch 6	73.89±0.37	5.18±0.01	0.58±0.02	17.92±0.00	90.8	715
Batch 7	74.12±0.08	5.09±0.04	0.45±0.00	18.05±0.14	92.9	701
				Average	92.4	696
				SD	1.18	20.9
				Min	90.6	651
				Max	93.5	715

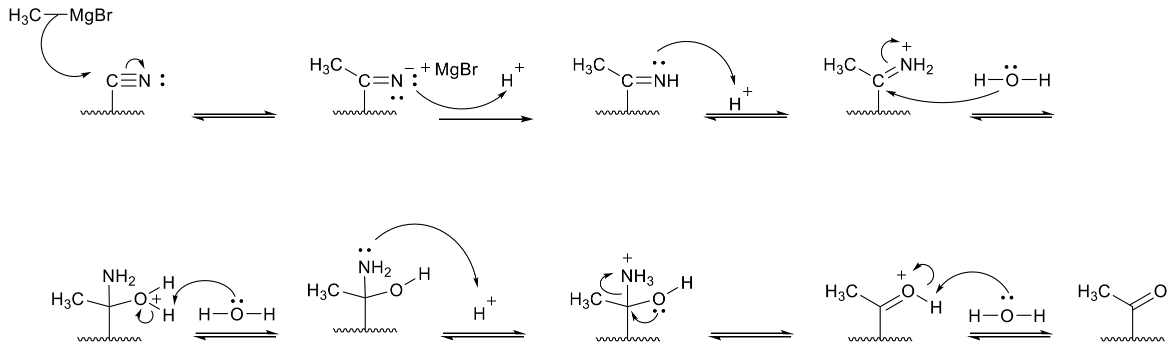

Supplementary Figure 9: The mechanism for the ketone formation.

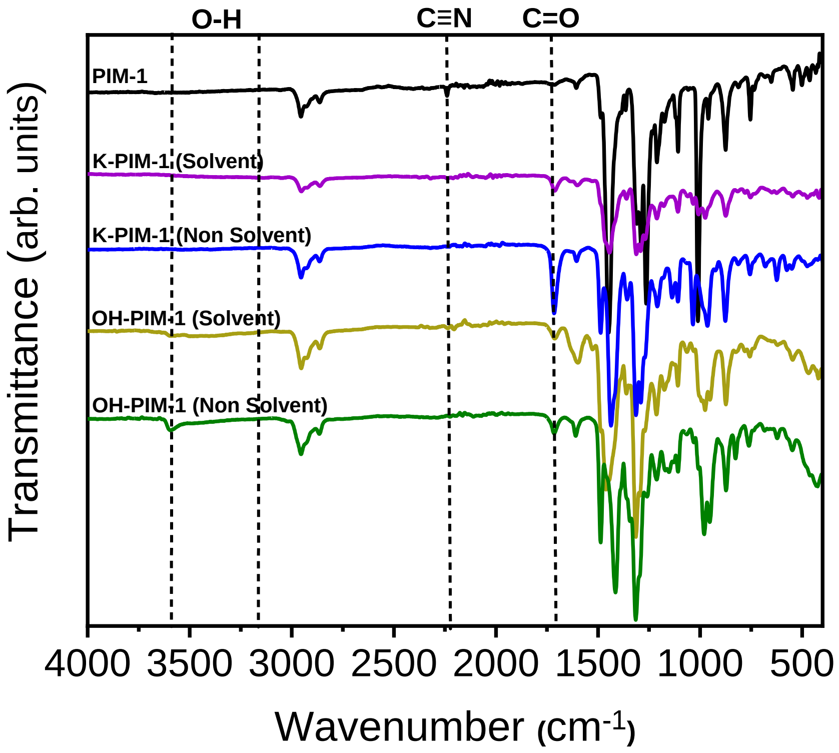

Supplementary Figure 10: FT-IR spectra of PIM-1, K-PIM-1, and OH-PIM-1 compared between the solvent and non-solvent methods.

Supplementary Table 3: Measurement of magnesium content by ICP-MS.

Sample	Avg. Mg (%)
PIM-1 (control)	0.000305 ± 0.0000073
K-PIM-1 (solvent method)	0.000294 ± 0.0000053
K-PIM-1 (non-solvent method)	0.000301 ± 0.0000015

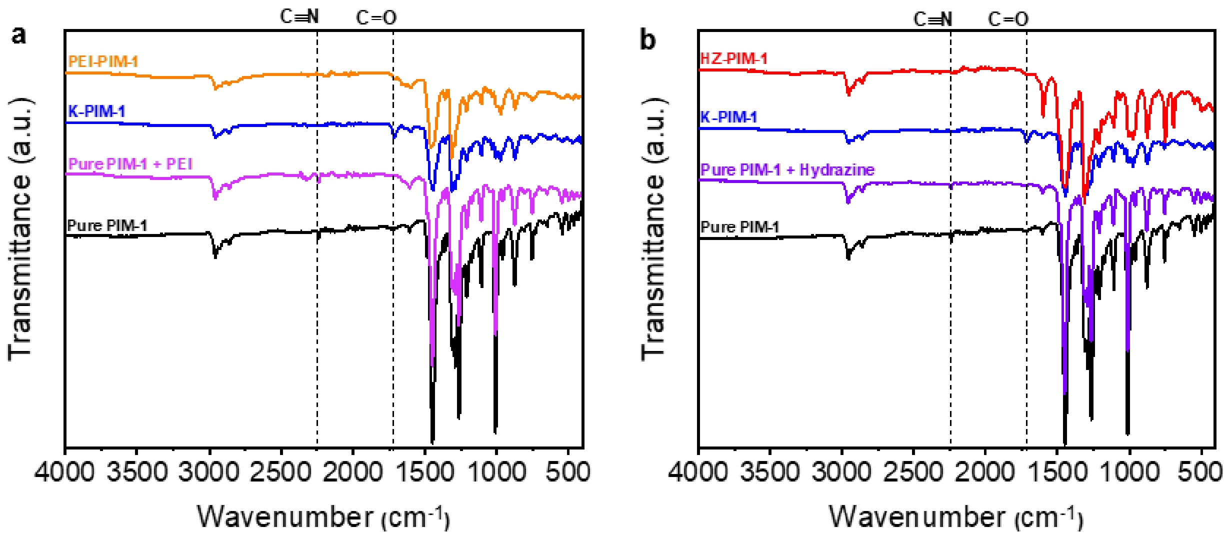

Supplementary Figure 11: FT-IR spectra to prove that PIM-1 was not able to create chemical bonding with amine and hydrazine if without K-PIM-1 as a media. **a** PEI-PIM-1 and **b** HZ-PIM-1 under Schiff's base reaction.

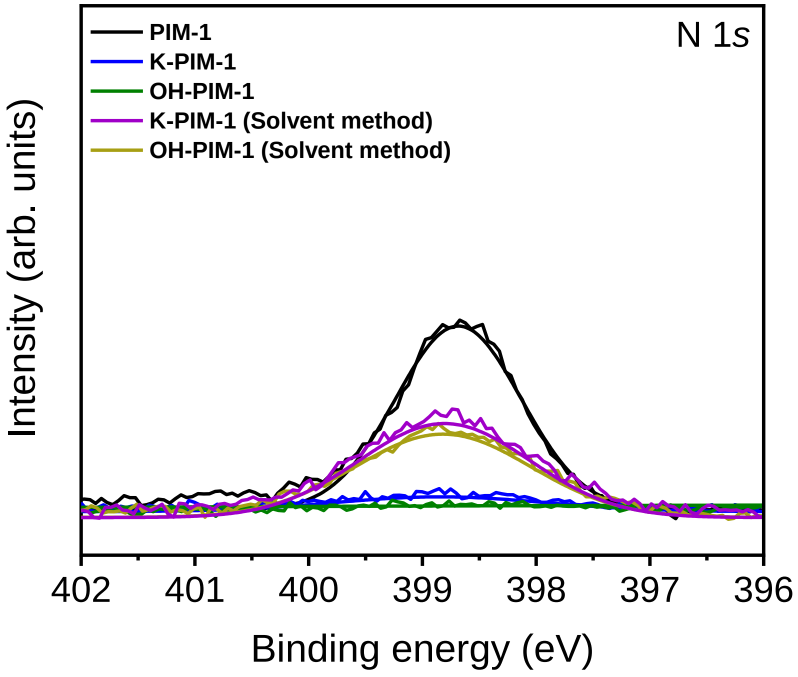

Supplementary Figure 12: XPS N1*s* for PIM-1, K-PIM-1 (solvent method), K-PIM-1 (non-solvent method), OH-PIM-1 (solvent method), and OH-PIM-1 (non-solvent method).

Supplementary Table 4: Elemental (CHNO) analysis of K-PIM-1 and OH-PIM-1 compared between the solvent and non-solvent methods.

Materials	%C		%H		%N		%O		% Conversion
	Theo.	Exp.	Theo.	Exp.	Theo.	Exp.	Theo.	Exp.	
PIM-1	75.64	74.61±0.00	4.38	4.33±0.02	6.08	6.30±0.02	13.90	13.07±0.14	-
K-PIM-1 (solvent method)	75.29	73.51±0.14	5.30	5.00±0.01	0.00	2.78±0.02	19.41	15.47±0.00	55.9
K-PIM-1	75.29	73.86±0.07	5.30	5.23±0.00	0.00	0.41±0.00	19.41	18.09±0.01	93.5
OH-PIM-1 (solvent method)	75.26	75.06±0.41	6.51	6.43±0.03	0.00	1.77±0.02	18.23	14.38±0.01	71.9
Further rxn-OH-PIM-1* (solvent method)	75.26	72.77±0.12	6.51	6.09±0.05	0.00	1.71±0.01	18.23	14.55±0.04	72.9
OH-PIM-1	75.26	74.13±0.02	6.51	6.09±0.08	0.00	0.20±0.00	18.23	17.68±0.36	96.8

*OH-PIM-1 was further reacted with the Grignard reagent using solvent method. So, this material was passed the third time of modification.

Supplementary Table 5: Comparison of surface composition obtained by XPS survey scan for PIM-1 and derivatives.

Material	%C	%N	%O	%Conversion
PIM-1	76.73	7.33	15.93	-
K-PIM-1	79.29	0.33	20.38	95.5
K-PIM-1 (solvent method)	77.36	3.07	19.59	58.1
OH-PIM-1	85.59	0	19.41	100.0
OH-PIM-1 (solvent method)	79.34	2.03	18.63	72.3
K-PIM-1 (EtMgBr)	79.99	0.36	19.66	95.1
K-PIM-1 (*t*-BuMgCl)	84.21	3.57	12.22	51.3
K-PIM-1 (*i*-BuMgBr)	81.13	0.64	18.23	91.3
K-PIM-1 (PhMgBr)	81.68	1.48	16.84	79.8

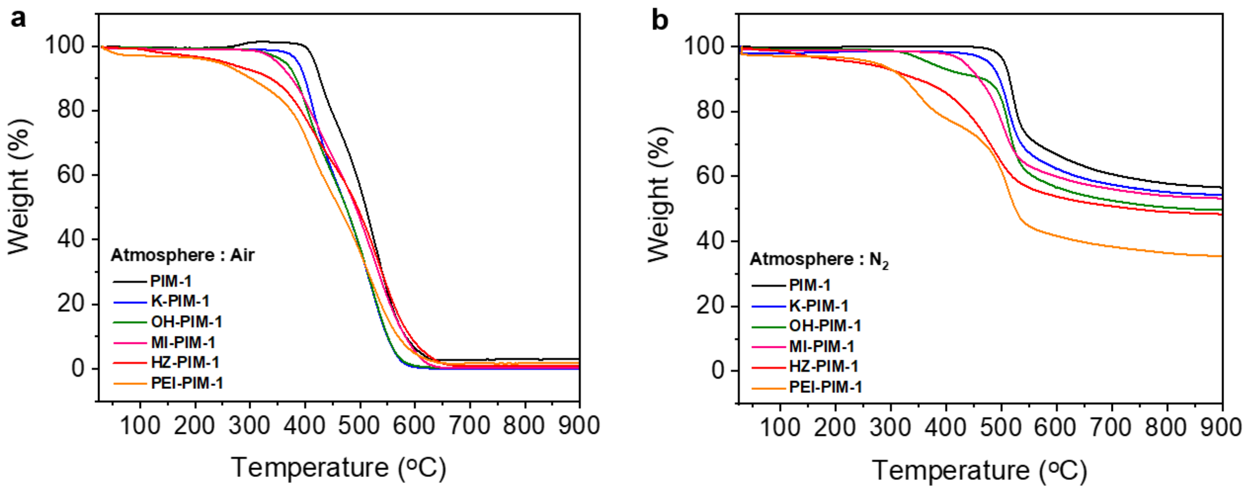

Supplementary Figure 13: Thermogravimetric analyses of PIM-1 and derivatives. The temperature was increased from 25 °C to 900 °C under **a** air and **b** N_2 atmospheres with a heating rate of 10 °C min^{-1}

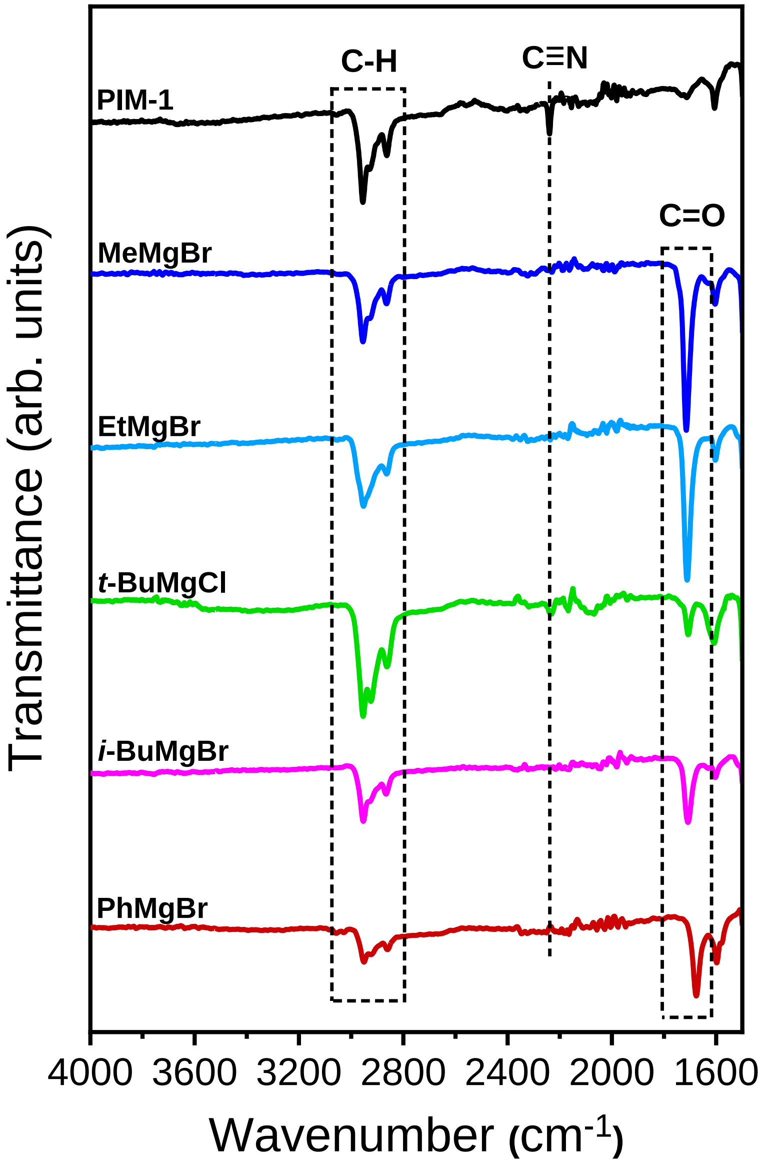

Supplementary Figure 14: FT-IR spectra of K-PIM-1 synthesized by a series of Grignard reagents via the non-solvent approach.

Supplementary Table 6: Elemental (CHNO) analysis of K-PIM-1 synthesized by a series of Grignard reagents via the non-solvent approach.

K-PIM-1	%C		%H		%N		%O		%Conversion	SA_{BET} (m^2 g^{-1})
	Theo.	Exp.	Theo.	Exp.	Theo.	Exp.	Theo.	Exp.		
MeMgBr	75.29	74.12±0.76	5.30	5.09±0.04	0	0.45±0.00	19.41	18.05±0.14	92.9	701
EtMgBr	75.84	75.06±0.14	5.79	5.67±0.32	0	0.38±0.02	18.37	17.06±0.05	94.0	573
t-BuMgCl	76.79	75.69±1.01	6.62	6.69±0.13	0	3.39±0.09	16.59	12.51±0.06	46.2	425
i-BuMgBr	76.79	75.74±0.04	6.62	6.42±0.03	0	1.11±0.01	16.59	15.11±0.02	82.4	346
PhMgBr	79.60	78.87±0.29	4.89	5.92±0.05	0	2.90±0.01	15.52	10.70±0.04	54.0*	9
PhMgBr	79.60	77.78±0.00	4.89	5.06±0.08	0	1.14±0.01	15.52	13.04±0.23	81.9	No surface area

* The additional post-modification for phenyl K-PIM-1 (PhMgBr) was conducted to compare surface area result with tert-butyl K-PIM-1 (*t*-BuMgCl) at half conversion.

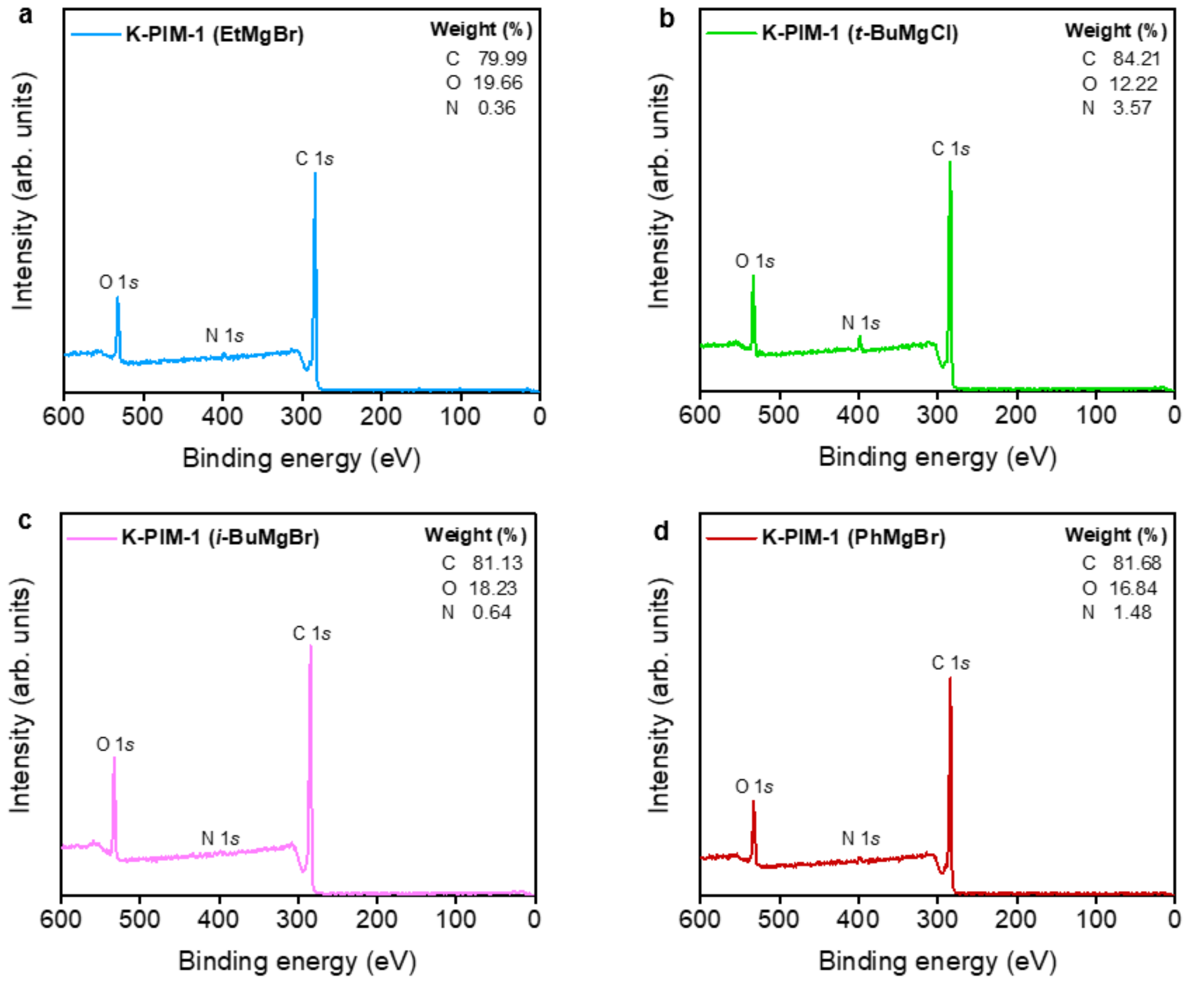

Supplementary Figure 15: XPS survey scan of **a** K-PIM-1 (EtMgBr), **b** K-PIM-1 (*t*-BuMgCl), **c** K-PIM-1 (*i*-BuMgBr), **d** K-PIM-1 (PhMgBr).

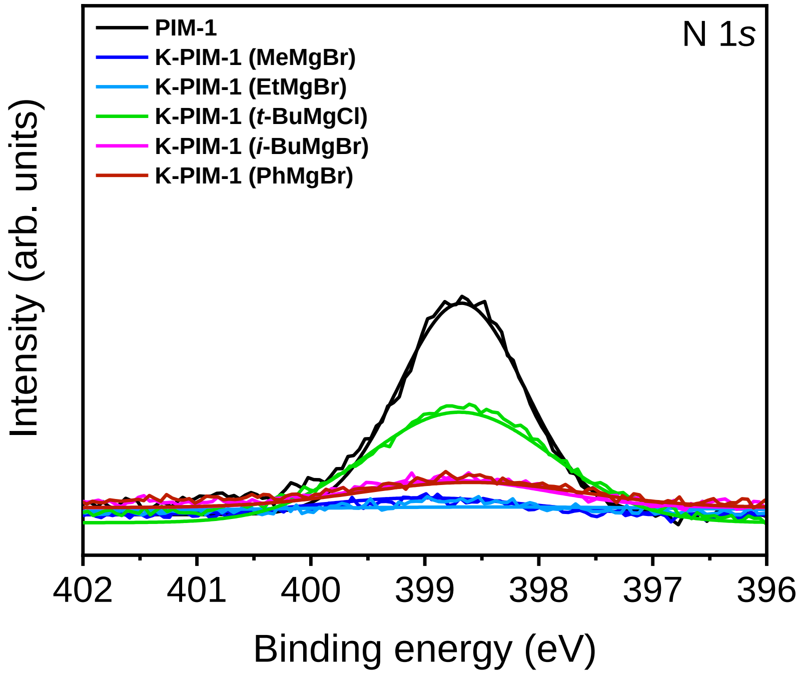

Supplementary Figure 16: XPS N1*s* for all K-PIM-1 derivatives.

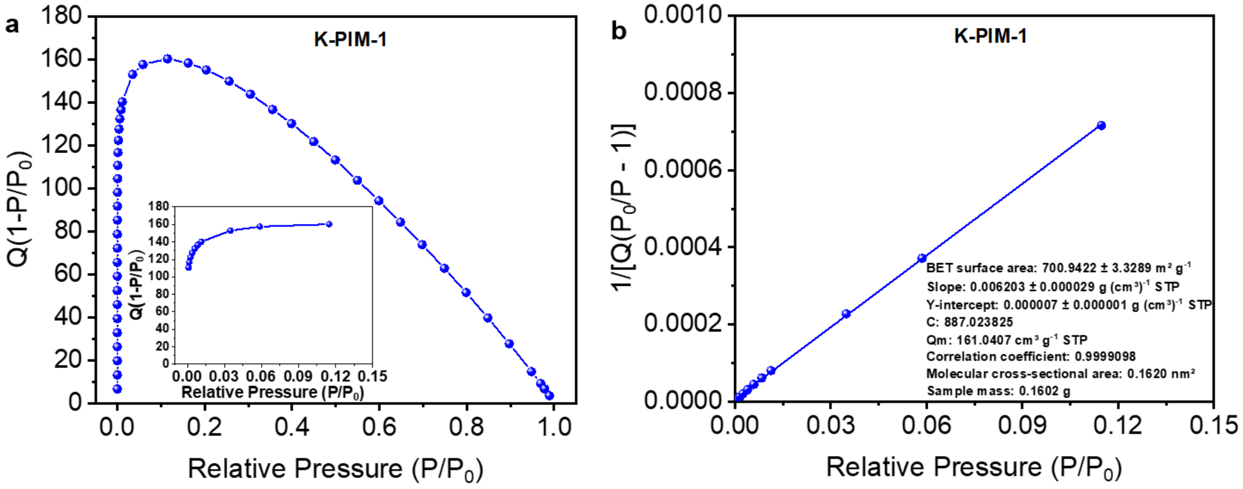

Supplementary Figure 17: a Calculated Rouquerol plot for K-PIM-1 along with the pressure ranges used for BET surface area calculations. **b** BET plot of K-PIM-1 obtained from N_2 adsorption isotherm at 77 K.

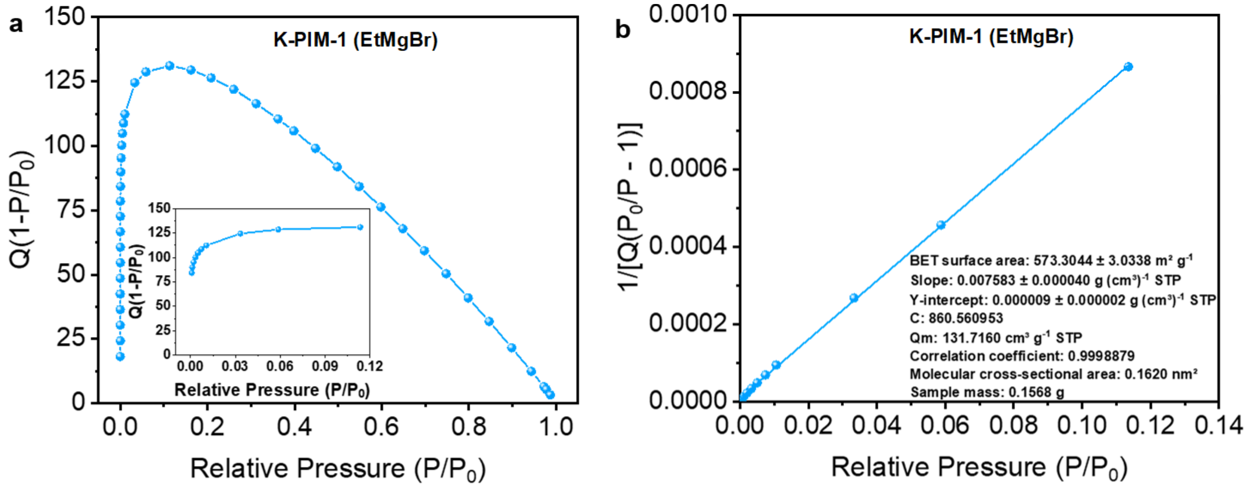

Supplementary Figure 18: a Calculated Rouquerol plot for K-PIM-1 (EtMgBr) along with the pressure ranges used for BET surface area calculations. **b** BET plot of K-PIM-1 (EtMgBr) obtained from N_2 adsorption isotherm at 77 K.

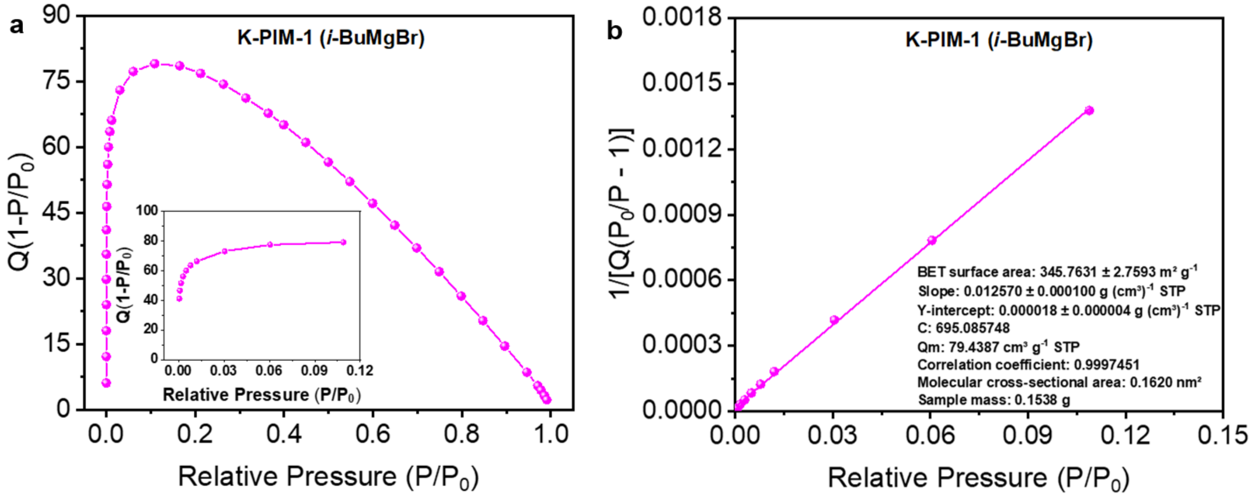

Supplementary Figure 19: a Calculated Rouquerol plot for K-PIM-1 (*i*-BuMgBr) along with the pressure ranges used for BET surface area calculations. **b** BET plot of K-PIM-1 (*i*-BuMgBr) obtained from N_2 adsorption isotherm at 77 K.

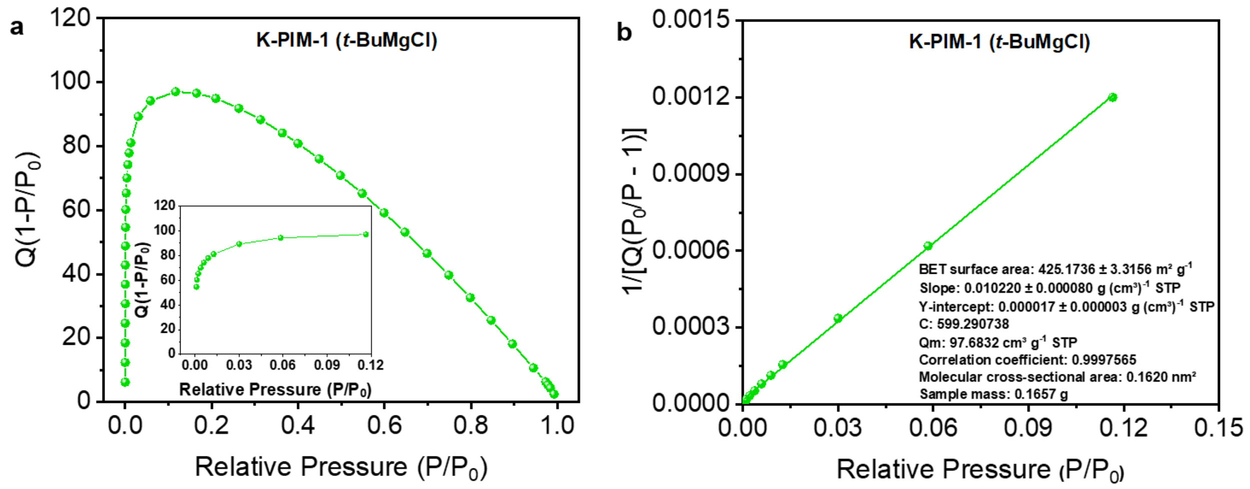

Supplementary Figure 20: a Calculated Rouquerol plot for K-PIM-1 (*t*-BuMgCl) along with the pressure ranges used for BET surface area calculations. **b** BET plot of K-PIM-1 (*t*-BuMgCl) obtained from N_2 adsorption isotherm at 77 K.

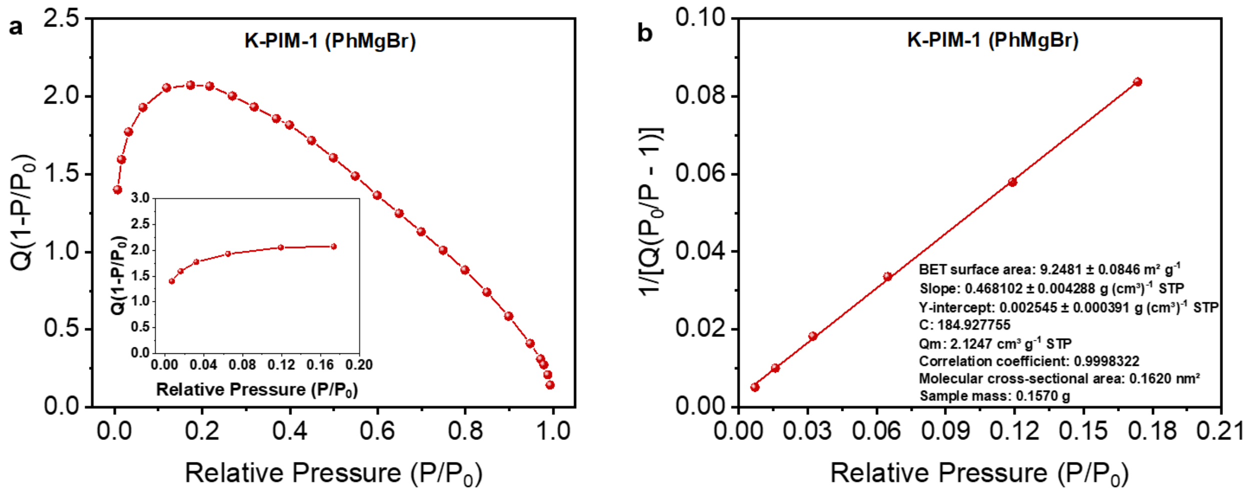

Supplementary Figure 21: a Calculated Rouquerol plot for K-PIM-1 (PhMgBr) along with the pressure ranges used for BET surface area calculations. **b** BET plot of K-PIM-1 (PhMgBr) obtained from N_2 adsorption isotherm at 77 K.

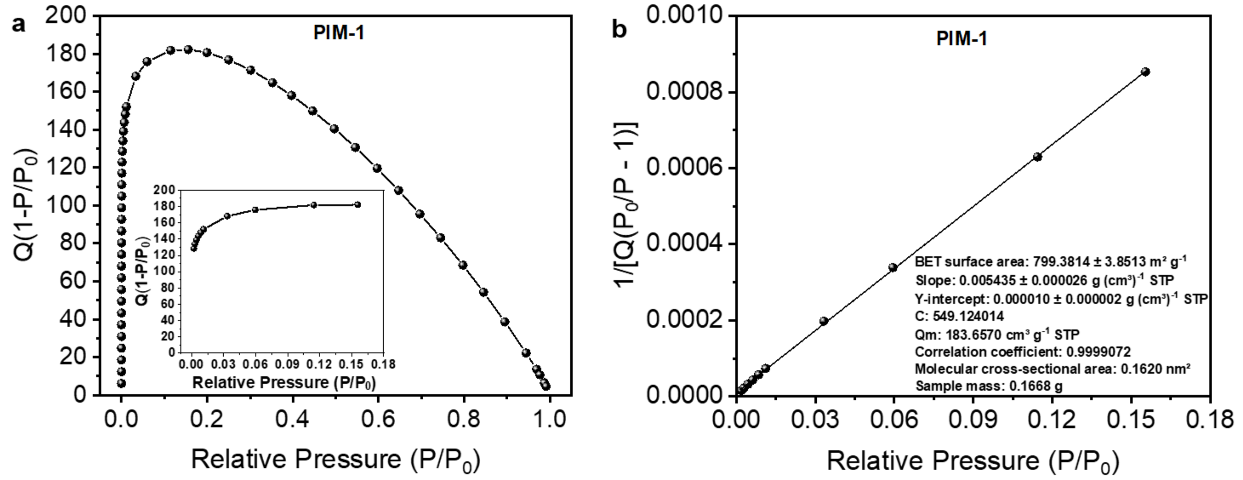

Supplementary Figure 22: a Calculated Rouquerol plot for PIM-1 along with the pressure ranges used for BET surface area calculations. **b** BET plot of PIM-1 obtained from N_2 adsorption isotherm at 77 K.

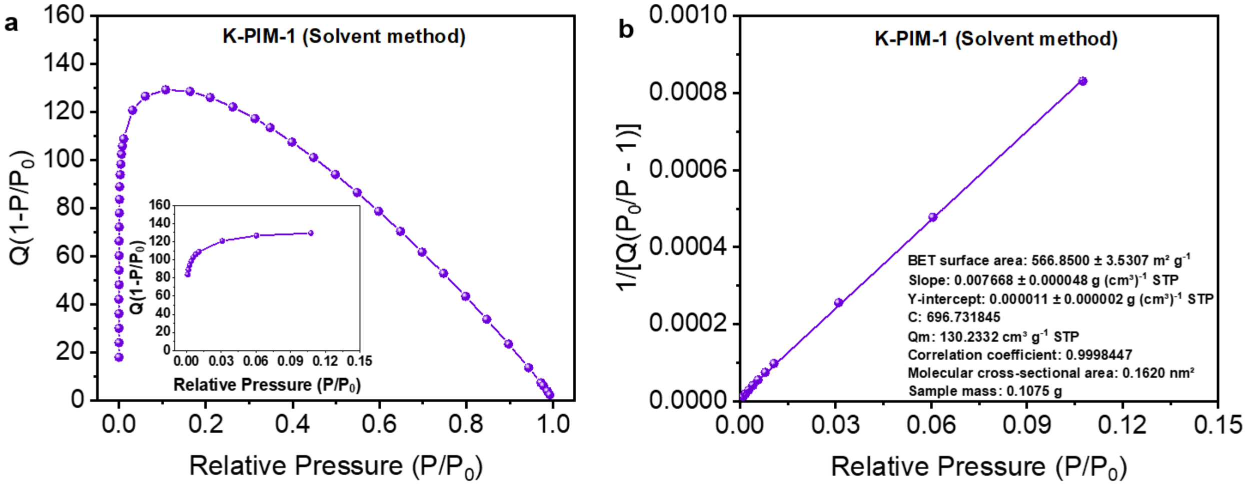

Supplementary Figure 23: a Calculated Rouquerol plot for K-PIM-1 (solvent method) along with the pressure ranges used for BET surface area calculations. **b** BET plot of K-PIM-1 (solvent method) obtained from N_2 adsorption isotherm at 77 K.

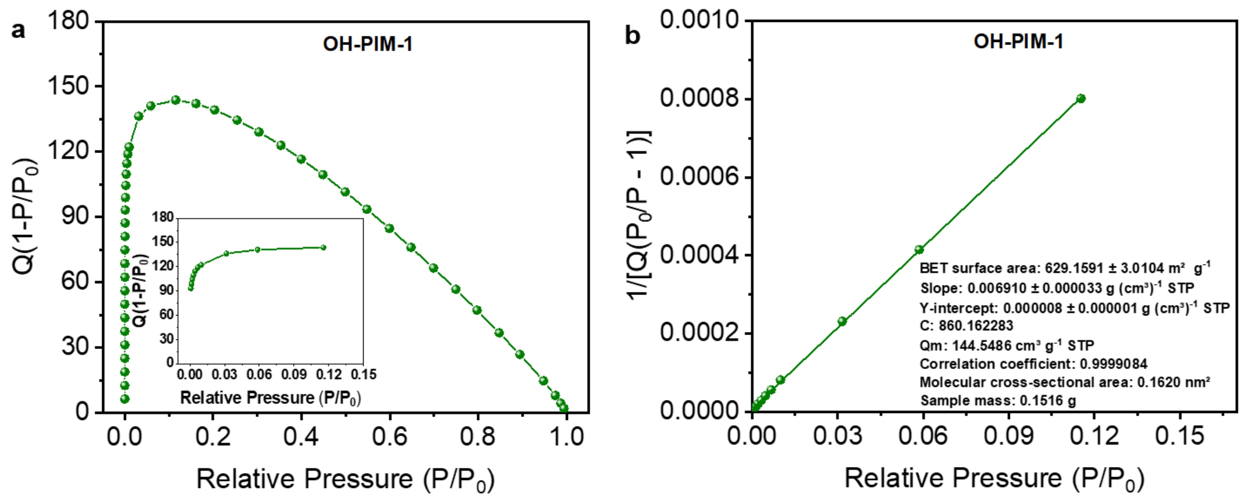

Supplementary Figure 24: a Calculated Rouquerol plot for OH-PIM-1 along with the pressure ranges used for BET surface area calculations. **b** BET plot of OH-PIM-1 obtained from N_2 adsorption isotherm at 77 K.

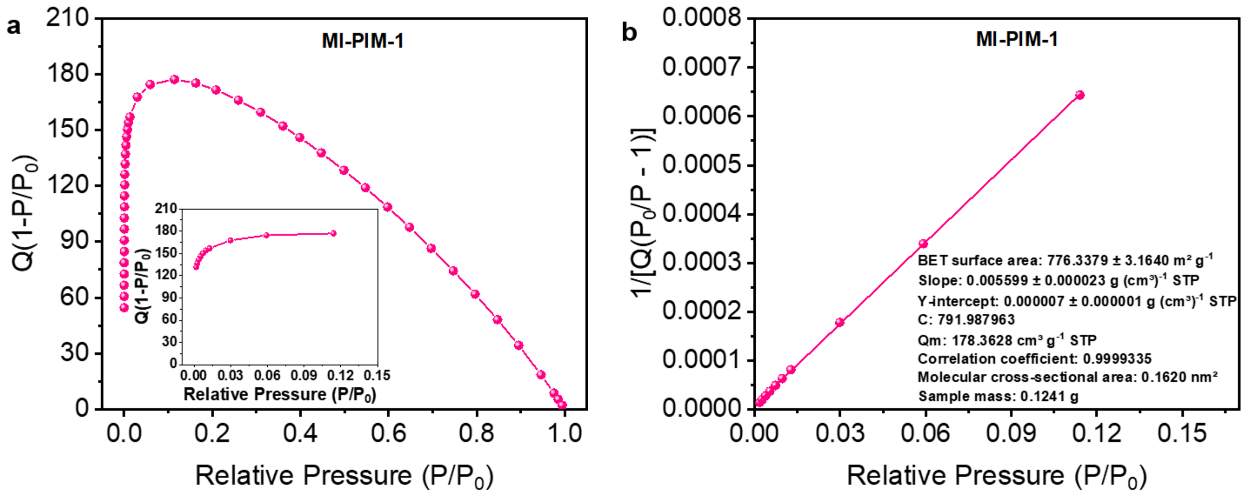

Supplementary Figure 25: a Calculated Rouquerol plot for MI-PIM-1 along with the pressure ranges used for BET surface area calculations. **b** BET plot of MI-PIM-1 obtained from N_2 adsorption isotherm at 77 K.

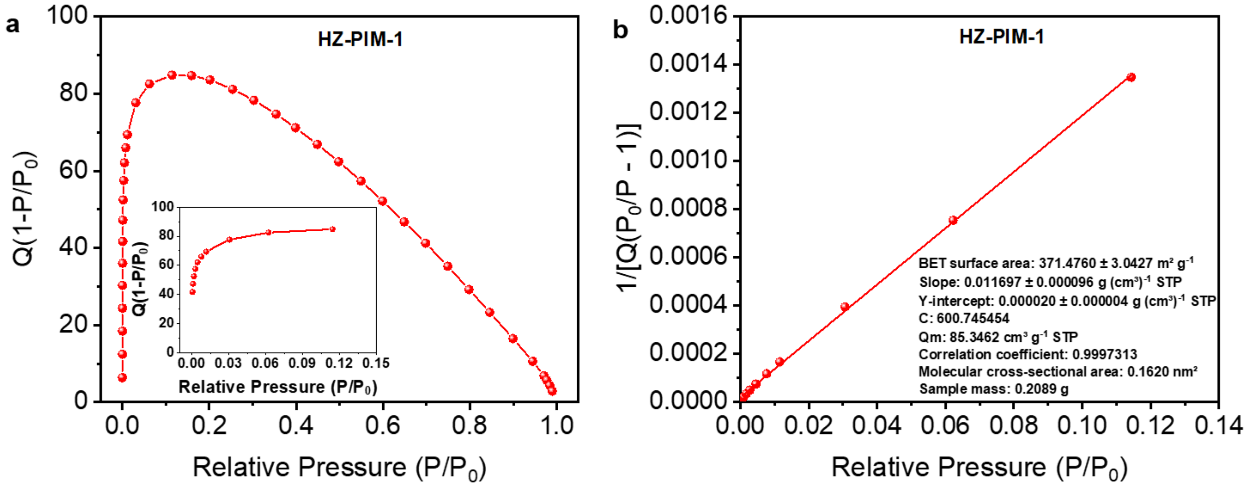

Supplementary Figure 26: a Calculated Rouquerol plot for HZ-PIM-1 along with the pressure ranges used for BET surface area calculations. **b** BET plot of HZ-PIM-1 obtained from N_2 adsorption isotherm at 77 K.

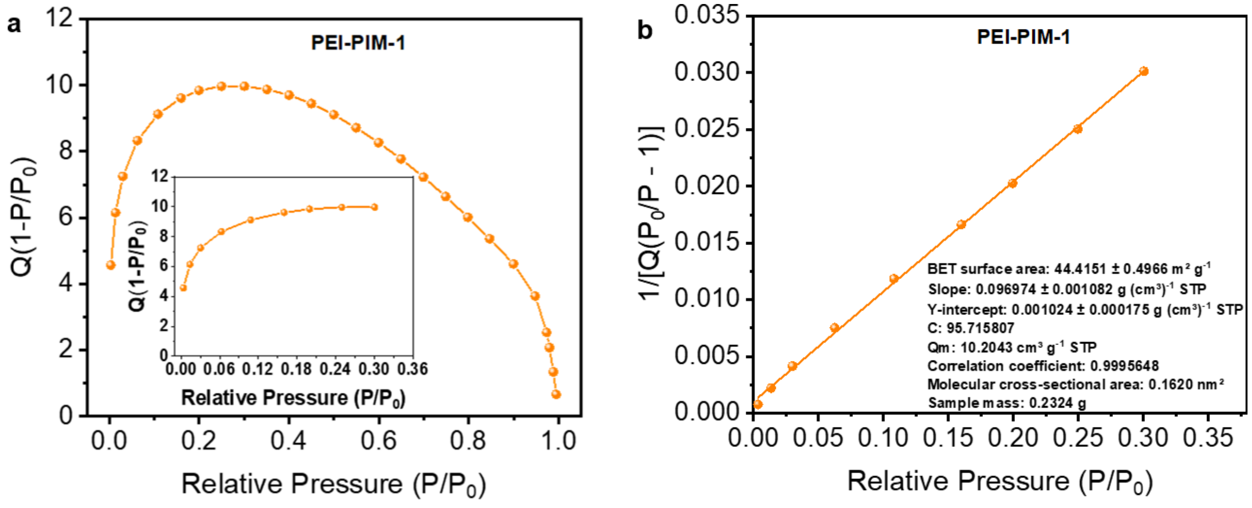

Supplementary Figure 27: a Calculated Rouquerol plot for PEI-PIM-1 along with the pressure ranges used for BET surface area calculations. **b** BET plot of PEI-PIM-1 obtained from N_2 adsorption isotherm at 77 K.

Supplementary Table 7: Comparison of the solvent and non-solvent methods' performance.

Parameter	Solvent method	Non-solvent method
%Conversion from PIM-1 to K-PIM-1	56	91-94
Surface area of K-PIM-1(m^2 g^{-1})	567	651-715
Surface area of OH-PIM-1(m^2 g^{-1})	460	629

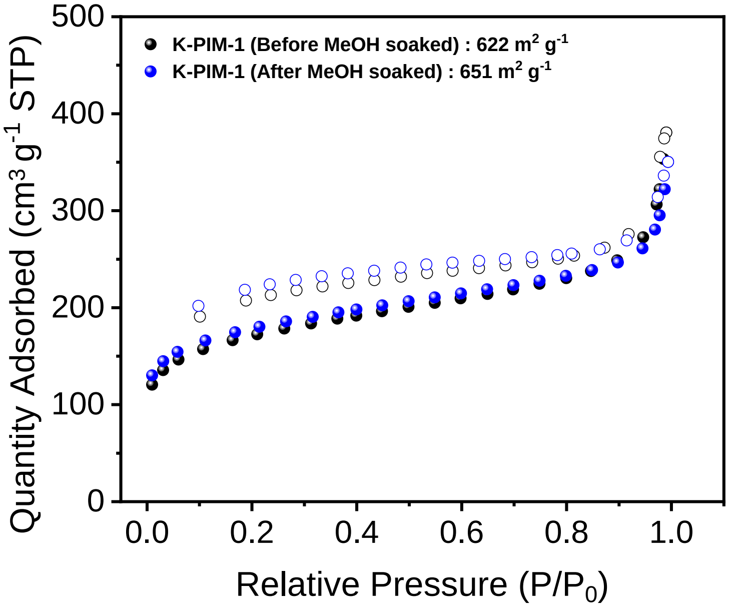

Supplementary Figure 28: N_2 physisorption isotherm at 77 K for K-PIM-1-non-solvent method compared between before and after MeOH treatment.

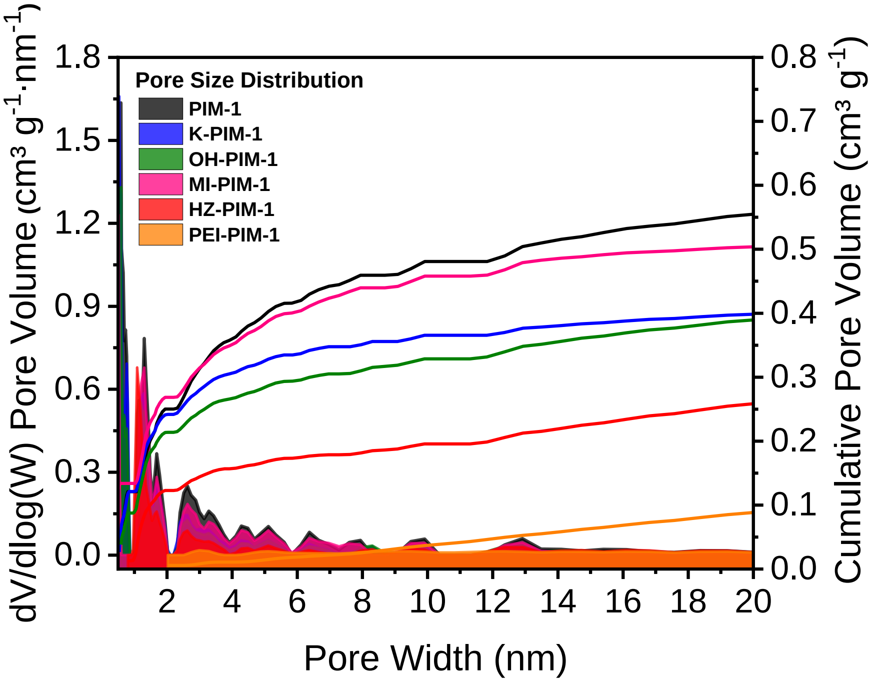

Supplementary Figure 29: Pore size distribution and cumulative pore volume of PIM-1 and derivatives.

Supplementary Table 8: Comparison of surface area and percent conversion of high-performance PIM-1 based modifications on cyano group in the literatures.

Materials	Surface area [m^2 g^{-1}]	%Conversion of cyano group	Reference
Methylimine-PIM-1 (MI-PIM-1)	776	Up to 94[a§]	This work
Ketone PIM-1 (K-PIM-1)	Up to 715	Up to 94[a]	This work
Alcohol-PIM-1 (OH-PIM-1)	629	97[a]	This work
Amidoxime-PIM-1 (AO-PIM-1)	577	NA	Our group's report
Amide-PIM-1 (PIM-$CONH_2$-24h)	527	95[b]	Yanaranop P. et al.[7]
Hydrazone-PIM-1 (HZ-PIM-1)	371	Up to 94[a§]	This work
Carboxylate-PIM-1 (PIM-COOH-36h)	284	94[d]	Jeon J. W. et al.[8]
Thioamide-PIM-1	263	80[c]	Mason C.R. et al.[9]
Tetrazole-PIM-1 (TZPIM-3)	30	100[e]	Du N.Y. et al.[10]
Thiophenyl- PIM-1 (PIM-B)	24.5	NA	Xu J.W. et al.[11]
Thiolethyl- PIM-1 (PIM-G)	18	NA	Xu J.W. et al.[11]
Diethanolamine-PIM-1 (DT150-24)	13	88[a]	Satilmis B et al.[12]
Ethanolamine-PIM-1 (ES120-24)	10	81[a]	Satilmis B et al.[12]
Amine-PIM-1	Isotherm could not be obtained	>90[f]	Mason C. R. et al.[13]
Methyl Tetrazole-PIM-1	NA	100	Du N.Y. et al.[14]

The surface area was measured from powder form. The reported values were obtained from the best conversion, which revealed the surface area data.

[a] The conversion was calculated from elemental analysis.

[b] The ratio between the integration of the -CN stretching vibration peak and the -CH stretching vibration peaks (Characterized from FT-IR) in relation to those for PIM-1 was used to determine the approximate extent of nitrile conversion.

[c] The conversion of nitrile to thioamide was estimated by integrating the ^1H NMR peaks and from the weight percent of sulfur determined by elemental analysis.

[d] The conversion obtained from the combination of amide (2%) and carboxylic acid (92%) contents calculated from ^1H NMR Spectra of hydrolyzed PIMs.

[e] The conversion was calculated from the molar ratio of the tetrazole groups in the TZPIM to the total nitrile groups of PIM-1, which is based on the ^1H NMR calculation of the integration ratio of the methyl signal of a methylated derivative of TZPIM to the methyl signals of the ladder polymer backbone.

[f] The conversion was estimated from IR and NMR data.

[§] The conversion was based on the post-modification of the K-PIM-1 derivative.

Supplementary Table 9: Comparison of gas uptake properties of PIM-1 and derivatives.

Material	SA_{BET} [m^2 g^{-1}]	CO_2 uptake [mmol g^{-1}]			CO_2 $Q_{st,max}$ at 0.05 bar [kJ mol^{-1}]	CO_2/N_2 selectivity at 15:85			H_2 at 77 K [mmol g^{-1}]	CH_4 uptake at 1 bar [mmol g^{-1}]	CO_2/CH_4 Gas Selectivity at 50:50
		273 K [0.15/1bar]	298 K [0.15/1bar]	323 K [0.15/1bar]		273 K	298 K	323 K		273 K	273 K
PIM-1	799	0.82/2.40	0.32/1.31	0.17/0.83	28.9	50	27	27	4.82	0.69	9
K-PIM-1	701	0.65/2.14	0.27/1.22	0.11/0.64	30.1	38	47	23	4.65	0.61	8
OH-PIM-1	629	0.53/1.85	0.21/1.02	0.09/0.52	27.2	37	70	22	4.23	0.53	7
MI-PIM-1	776	0.64/2.24	0.28/1.28	0.13/0.70	26.9	29	18	13	5.31	0.69	7
HZ-PIM-1	371	0.31/1.00	0.12/0.54	0.04/0.26	19.5	50	53	16	2.92	0.30	8
PEI-PIM-1	44	1.39/2.34	0.94/1.70	0.57/1.17	44.5[a]	403	316	273	1.75	0.20	3066

[a]$Q_{st,max}$ under CO_2 loading (mmol g^{-1}) at 0.90 bar.

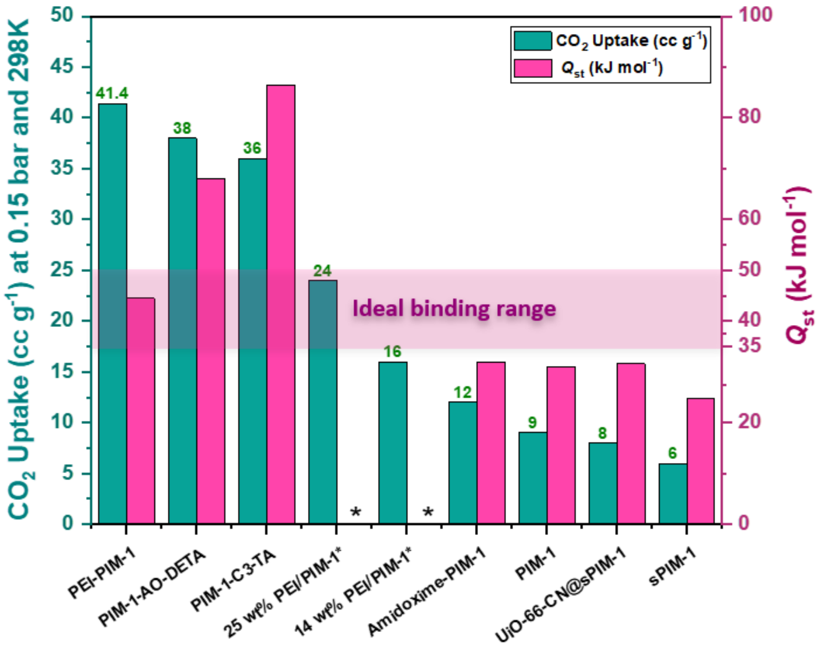

Supplementary Figure 30: Benchmarking CO_2 uptake at a low partial pressure versus Q_{st} values with other reported adsorbents.

Supplementary Table 10: Comparison of CO_2 capture performance of covalently tethered PEI.

Materials	CO_2 adsorption			CO_2/N_2 at 25 °C	Q_{st} (kJ mol^{-1})	Reference
	Capacity (mmol g^{-1})	Temperature (°C)	Pressure			
PEI-PIM-1	1.70[a]	25	1.01 bar	316[h]	44.5	This work
PP-AM-PEI fiber	5.91[b]	25	-	-	-	Wu Q. et al.[15]
PEI-MIL-101-100	5.00[c]	25	1 bar	600[i]	-	Lin Y. et al.[16]
HG–PEI-1.98	4.13[d]	25	1 atm		68[k]	Liu F. et al.[17]
PEI70@PGD-H	4.17[a]	25	1 atm	73.3	48..4[l]	Zhu J. et al.[18]
Porous PS-PEI	3.46[a]	40	1 bar	26.9[j]	-	Liu Z. et al.[19]
PEI50/AlSiO_2-TPI	2.51[e]	50	-	-	-	Kolle J. M. et al.[20]
GMA-PEI (NUT-10)	2.09[a]	0	1 bar	308	49	Mane S. et al.[21]
PEI/SiO_2	1.98[f]	60	-		66.2[m]	Min K. et al.[22]
EB-PEI/SiO_2	1.62[f]	60	-		80.5[m]	Min K. et al.[22]
CNT-PEI	0.98[g]	70	~1 atm	-	-	Zhou Z. et al.[23]
PEI grafted MCM-41(M1)	<1[a]	30	1.01 bar	-	-	Kassab H. et al.[24]

[a] Adsorption isotherm (Pure CO_2).

[b] CO_2 breakthrough experiment (10%CO_2/N_2).

[c] The CO_2 adsorption kinetics of the probe gas CO_2 were also measured using volumetric technique by the apparatus from SETARAM France (PCTpro-E&E).

[d] The CO_2 capture experiment was performed under anhydrous conditions using a TGA/DSC thermogravimetric analyzer (10% CO_2 balanced with argon).

[e] Adsorption capacity was determined by TGA based on weight gain after exposure of the dry material to a 15% CO_2/N_2

[f] TGA-MS (15% CO_2, 10% H_2O, N_2 balance).

[g] The CO_2 capture was conducted in a column flow reactor system (Pure CO_2).

[h] CO_2/N_2 at 15/85.

[i] 0.15 bar CO_2 and 0.75 bar N_2.

[j] CO_2 separation capacity, using a flow of 14% (v/v) CO_2 mixed with N_2.

[k] The adsorption enthalpies, calculated from the DSC heat flow profiles during the adsorption process.

[l] CO_2 desorption heat determined by DSC.

[m] Heat of CO_2 adsorption by DSC.

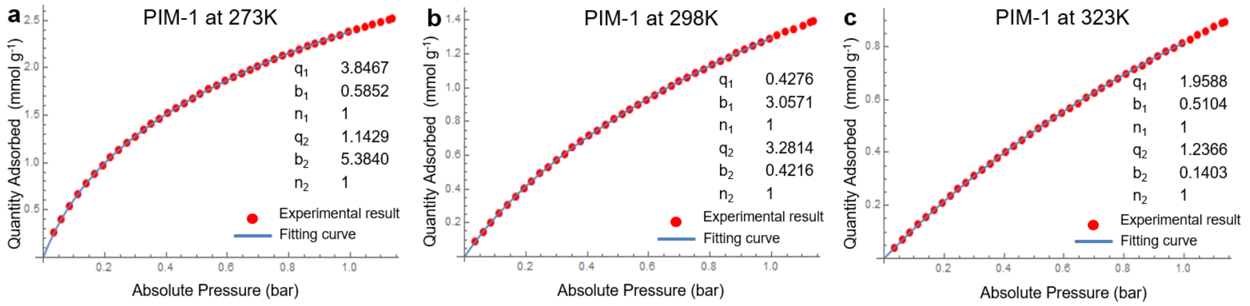

Supplementary Figure 31: CO_2 adsorption isotherms of PIM-1 at **a** 273 K, **b** 298 K, **c** 323 K fit to the Dual-Site Langmuir-Freundlich (DSLF) model by Wolfram Mathematica.

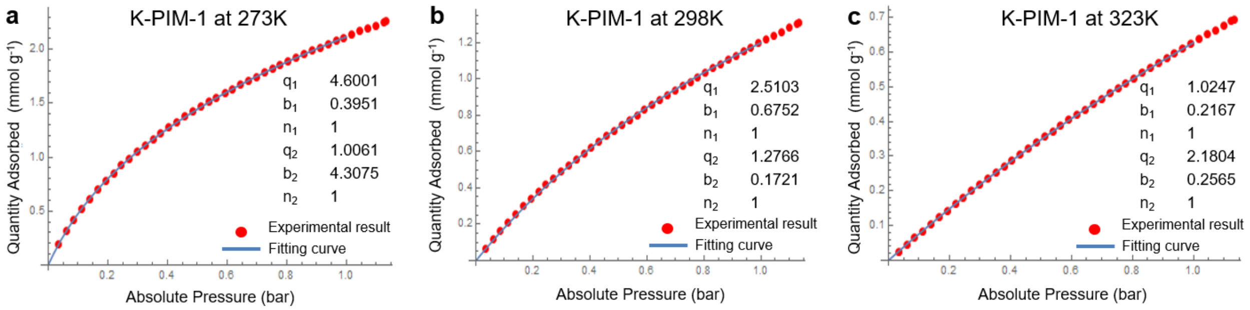

Supplementary Figure 32: CO_2 adsorption isotherms of K-PIM-1 at **a** 273 K, **b** 298 K, **c** 323 K fit to the Dual-Site Langmuir-Freundlich (DSLF) model by Wolfram Mathematica.

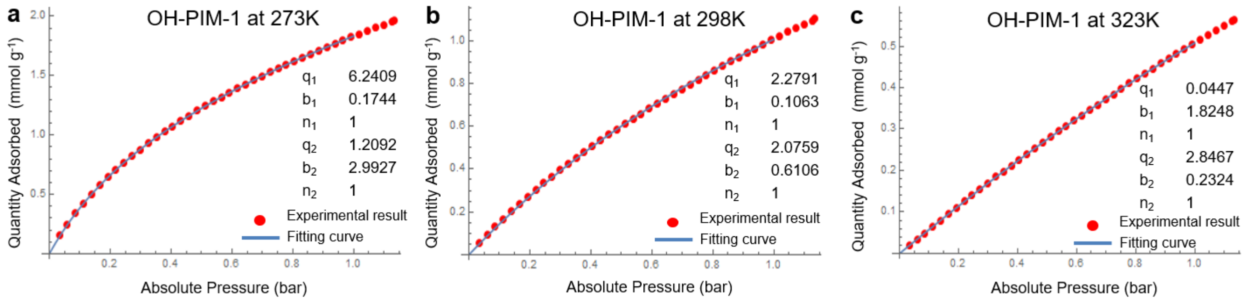

Supplementary Figure 33: CO_2 adsorption isotherms of OH-PIM-1 at **a** 273 K, **b** 298 K, **c** 323 K fit to the Dual-Site Langmuir-Freundlich (DSLF) model by Wolfram Mathematica.

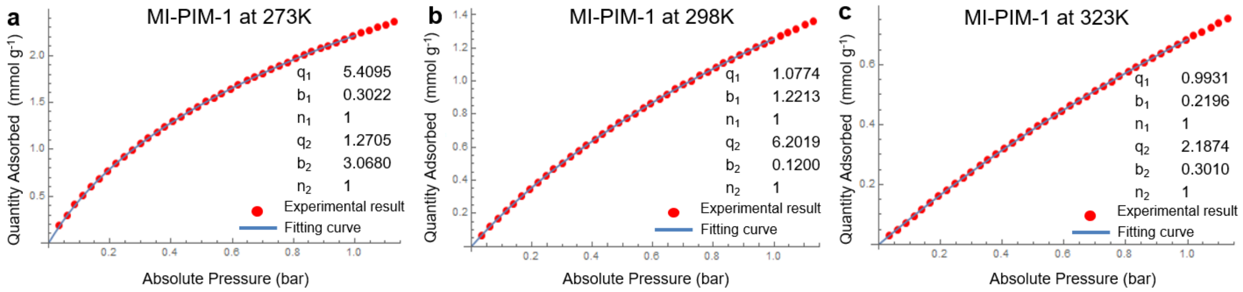

Supplementary Figure 34: CO_2 adsorption isotherms of MI-PIM-1 at **a** 273 K, **b** 298 K, **c** 323 K fit to the Dual-Site Langmuir-Freundlich (DSLF) model by Wolfram Mathematica.

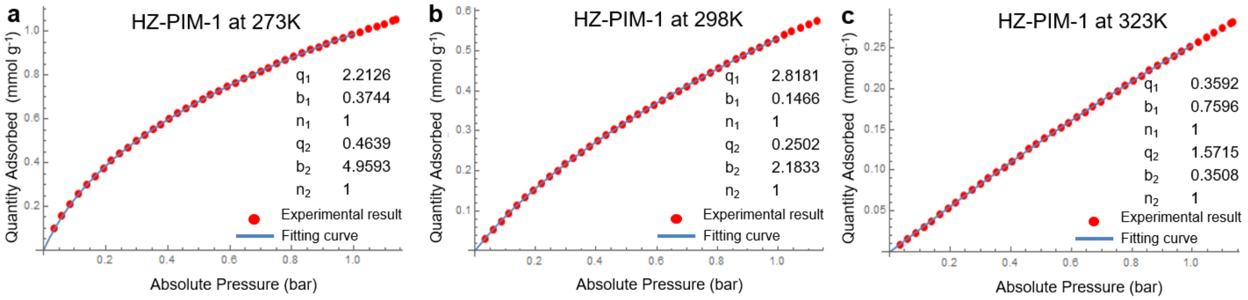

Supplementary Figure 35: CO_2 adsorption isotherms of HZ-PIM-1 at **a** 273 K, **b** 298 K, **c** 323 K fit to the Dual-Site Langmuir-Freundlich (DSLF) model by Wolfram Mathematica.

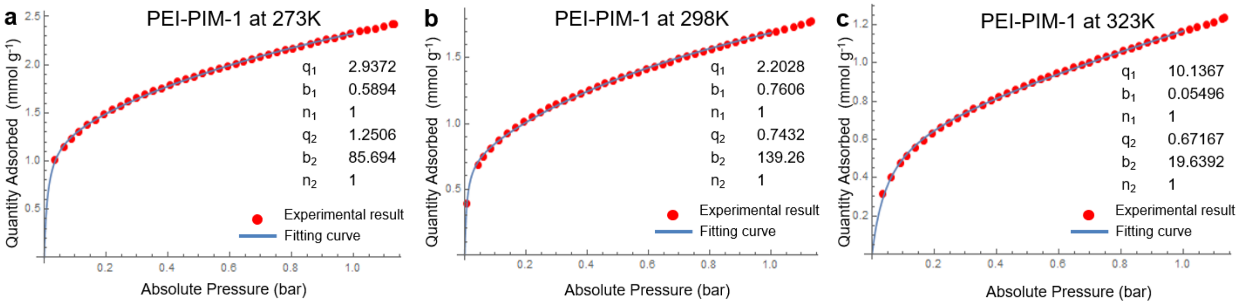

Supplementary Figure 36: CO_2 adsorption isotherms of PEI-PIM-1 at **a** 273 K, **b** 298 K, **c** 323 K fit to the Dual-Site Langmuir-Freundlich (DSLF) model by Wolfram Mathematica.

Supplementary Table 11: Comparison of Q_{st} values with three temperatures with Wolfram Mathematica for PIM-1, K-PIM-1, and OH-PIM-1.

PIM-1					K-PIM-1					OH-PIM-1				
CO_2 loading (mmol g^{-1})	P@273	P@298	P@323	Q_{st} (kJ mol^{-1})	CO_2 loading (mmol g^{-1})	P@273	P@298	P@323	Q_{st} (kJ mol^{-1})	CO_2 loading (mmol g^{-1})	P@273	P@298	P@323	Q_{st} (kJ mol^{-1})
0.05	0.006	0.019	0.043	28.85	0.05	0.008	0.027	0.065	30.13	0.05	0.011	0.034	0.069	27.18
0.1	0.012	0.040	0.089	28.79	0.1	0.017	0.054	0.132	29.99	0.1	0.022	0.069	0.142	27.18
0.15	0.019	0.061	0.136	28.72	0.15	0.026	0.082	0.201	29.84	0.15	0.034	0.105	0.218	27.16
0.2	0.026	0.084	0.185	28.66	0.2	0.036	0.112	0.273	29.69	0.2	0.047	0.142	0.298	27.13
0.25	0.034	0.108	0.236	28.60	0.25	0.046	0.142	0.347	29.55	0.25	0.060	0.181	0.381	27.10
0.3	0.042	0.134	0.289	28.54	0.3	0.057	0.173	0.424	29.41	0.3	0.074	0.222	0.468	27.05
0.35	0.050	0.161	0.345	28.48	0.35	0.068	0.206	0.503	29.27	0.35	0.089	0.264	0.559	27.00
0.4	0.058	0.190	0.403	28.43	0.4	0.080	0.240	0.585	29.13					
0.45	0.067	0.220	0.463	28.37	0.45	0.093	0.274	0.671	29.00					
0.5	0.077	0.251	0.527	28.32	0.5	0.106	0.311	0.759	28.87					
0.55	0.087	0.284	0.593	28.28	0.55	0.120	0.348	0.851	28.75					
0.6	0.097	0.319	0.662	28.23										
0.65	0.108	0.355	0.735	28.20										
0.7	0.120	0.393	0.811	28.16										
0.75	0.132	0.433	0.891	28.14										
0.8	0.144	0.474	0.976	28.11										

Supplementary Table 12: Comparison of Q_{st} values with three temperatures with Wolfram Mathematica for MI-PIM-1, HZ-PIM-1, and PEI-PIM-1.

MI-PIM-1					HZ-PIM-1					PEI-PIM-1				
CO_2 loading (mmol g^{-1})	P@273	P@298	P@323	Q_{st} (kJ mol^{-1})	CO_2 loading (mmol g^{-1})	P@273	P@298	P@323	Q_{st} (kJ mol^{-1})	CO_2 loading (mmol g^{-1})	P@273	P@298	P@323	Q_{st} (kJ mol^{-1})
0.05	0.009	0.025	0.058	26.94	0.05	0.017	0.056	0.063	19.50	0.05	0.0005	0.001	0.004	29.91
0.1	0.019	0.051	0.118	26.88	0.1	0.036	0.119	0.129	19.05	0.1	0.001	0.001	0.008	30.43
0.15	0.029	0.077	0.180	26.82	0.15	0.058	0.190	0.199	18.60	0.15	0.002	0.002	0.014	31.00
0.2	0.039	0.105	0.244	26.76	0.2	0.082	0.270	0.274	18.15	0.2	0.002	0.003	0.020	31.64
0.25	0.050	0.134	0.310	26.71	0.25	0.109	0.358	0.354	17.69	0.25	0.003	0.004	0.027	32.35
0.3	0.061	0.164	0.379	26.66	0.3	0.140	0.453	0.440	17.25	0.3	0.004	0.005	0.036	33.14
0.35	0.073	0.196	0.450	26.61	0.35	0.174	0.557	0.531	16.82	0.35	0.004	0.006	0.047	34.04
0.4	0.085	0.228	0.523	26.56	0.4	0.212	0.669	0.629	16.41	0.4	0.005	0.008	0.061	35.03
0.45	0.098	0.262	0.600	26.52						0.45	0.006	0.010	0.078	36.13
0.5	0.111	0.297	0.679	26.48						0.5	0.007	0.013	0.100	37.34
0.55	0.125	0.333	0.761	26.45						0.55	0.009	0.017	0.127	38.63
0.6	0.140	0.371	0.847	26.42						0.6	0.010	0.022	0.162	39.95
0.65	0.155	0.410	0.936	26.39						0.65	0.012	0.030	0.204	41.24
0.7	0.170	0.450	1.029	26.38						0.7	0.014	0.041	0.255	42.41
										0.75	0.016	0.056	0.314	43.37
										0.8	0.019	0.076	0.381	44.07
										0.85	0.022	0.100	0.453	44.45
										0.91	0.025	0.128	0.531	44.53

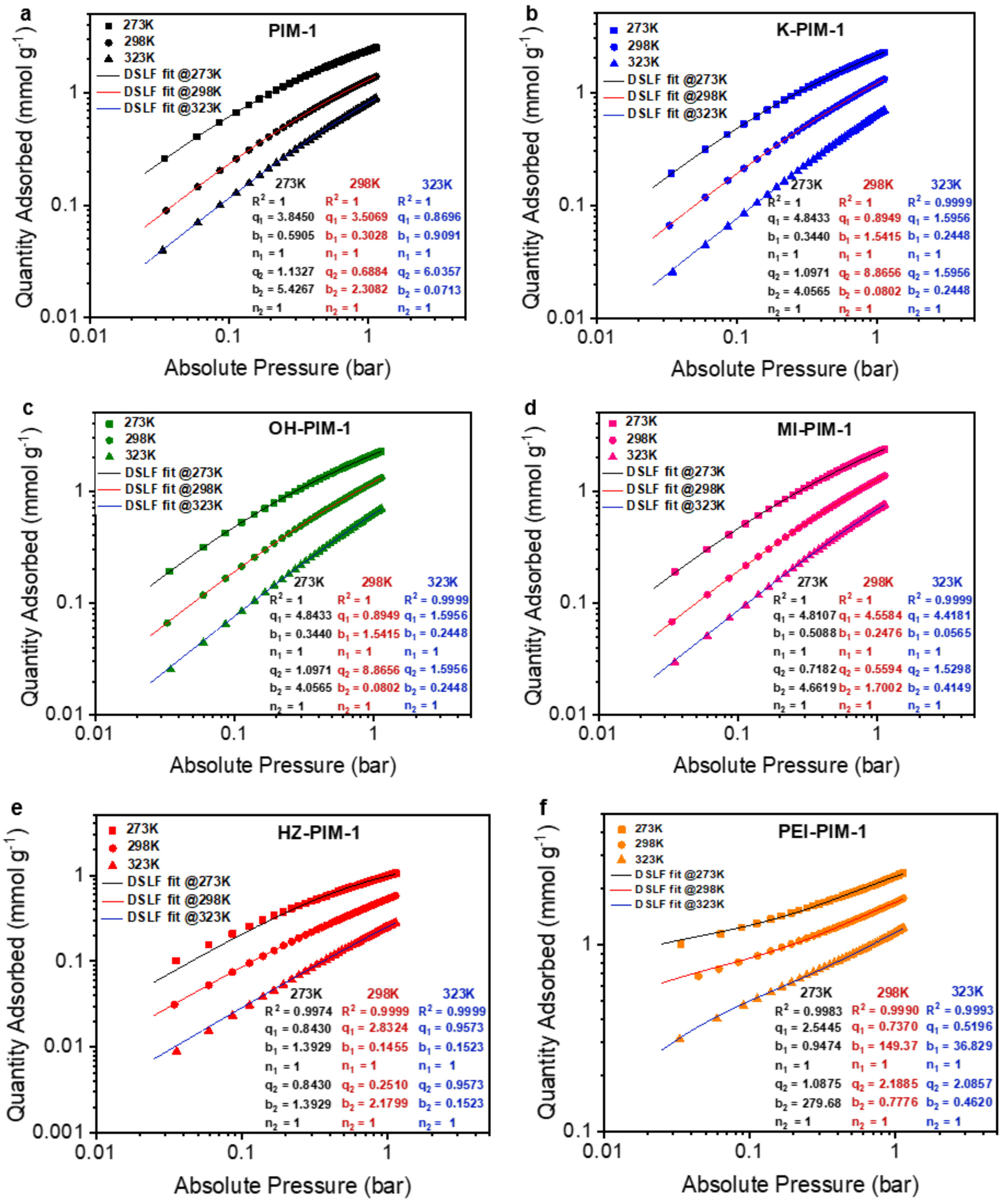

Supplementary Figure 37: Logarithmic-scale plots for Dual-Site Langmuir-Freundlich (DSLF) model fitting for CO_2 adsorption isotherms at 273 K, 298 K, and 323 K by OriginPro on **a** PIM-1, **b** K-PIM-1, **c** OH-PIM-1, **d** MI-PIM-1, **e** HZ-PIM-1, and **f** PEI-PIM-1 (An alternative approach).

Supplementary Table 13: Comparison of Q_{st} values with three temperatures with OriginPro for PIM-1, K-PIM-1, and OH-PIM-1.

PIM-1					K-PIM-1					OH-PIM-1				
CO_2 loading (mmol g^{-1})	P@273	P@298	P@323	Q_{st} (kJ mol^{-1})	CO_2 loading (mmol g^{-1})	P@273	P@298	P@323	Q_{st} (kJ mol^{-1})	CO_2 loading (mmol g^{-1})	P@273	P@298	P@323	Q_{st} (kJ mol^{-1})
0.05	0.006	0.019	0.042	28.39	0.05	0.008	0.025	0.065	29.99	0.05	0.0084	0.0245	0.0650	29.99
0.1	0.012	0.040	0.086	28.40	0.1	0.017	0.050	0.132	29.86	0.1	0.0172	0.0503	0.1322	29.86
0.15	0.019	0.062	0.133	28.41	0.15	0.026	0.077	0.201	29.73	0.15	0.0265	0.0773	0.2015	29.73
0.2	0.026	0.085	0.181	28.41	0.2	0.036	0.106	0.273	29.60	0.2	0.0362	0.1057	0.2732	29.60
0.25	0.034	0.109	0.232	28.41	0.25	0.046	0.136	0.347	29.47	0.25	0.0464	0.1355	0.3472	29.47
0.3	0.041	0.134	0.286	28.40	0.3	0.057	0.167	0.424	29.35	0.3	0.0572	0.1667	0.4239	29.35
0.35	0.050	0.161	0.342	28.39	0.35	0.068	0.199	0.503	29.22	0.35	0.0685	0.1994	0.5033	29.22
0.4	0.058	0.190	0.401	28.37	0.4	0.080	0.234	0.585	29.10					
0.45	0.067	0.219	0.462	28.35	0.45	0.093	0.269	0.671	28.97					
0.5	0.077	0.251	0.527	28.33	0.5	0.106	0.307	0.759	28.86					
0.55	0.087	0.284	0.594	28.30	0.55	0.120	0.345	0.851	28.74					
0.6	0.097	0.318	0.664	28.27										
0.65	0.108	0.355	0.737	28.23										
0.7	0.120	0.393	0.812	28.19										
0.75	0.132	0.432	0.891	28.14										
0.8	0.144	0.474	0.973	28.08										

Supplementary Table 14: Comparison of Q_{st} values with three temperatures with OriginPro for MI-PIM-1, HZ-PIM-1, and PEI-PIM-1.

MI-PIM-1					HZ-PIM-1					PEI-PIM-1				
CO_2 loading (mmol g^{-1})	P@273	P@298	P@323	Q_{st} (kJ mol^{-1})	CO_2 loading (mmol g^{-1})	P@273	P@298	P@323	Q_{st} (kJ mol^{-1})	CO_2 (mmol g^{-1})	P@273	P@298	P@323	Q_{st} (kJ mol^{-1})
0.05	0.009	0.025	0.058	27.45	0.05	0.022	0.056	0.176	30.36	0.05	0.0002	0.000	0.003	40.20
0.1	0.018	0.050	0.117	27.35	0.1	0.045	0.119	0.362	30.33	0.1	0.000	0.001	0.006	40.94
0.15	0.028	0.077	0.179	27.25	0.15	0.070	0.190	0.558	30.30	0.15	0.001	0.002	0.010	41.77
0.2	0.038	0.105	0.243	27.15	0.2	0.097	0.270	0.766	30.26	0.2	0.001	0.002	0.015	42.72
0.25	0.049	0.134	0.309	27.05	0.25	0.125	0.358	0.986	30.21	0.25	0.001	0.003	0.021	43.79
0.3	0.060	0.164	0.378	26.96	0.3	0.155	0.453	1.220	30.16	0.3	0.001	0.004	0.030	45.02
0.35	0.072	0.195	0.450	26.87	0.35	0.188	0.557	1.469	30.09	0.35	0.002	0.006	0.041	46.40
0.4	0.084	0.228	0.524	26.79	0.4	0.223	0.669	1.734	30.02	0.4	0.002	0.007	0.055	47.92
0.45	0.097	0.262	0.600	26.71						0.45	0.002	0.010	0.074	49.52
0.5	0.110	0.297	0.680	26.64						0.5	0.003	0.012	0.100	51.12
0.55	0.124	0.333	0.762	26.57						0.55	0.004	0.016	0.131	52.59
0.6	0.139	0.371	0.848	26.50						0.6	0.004	0.022	0.170	53.81
0.65	0.154	0.410	0.936	26.44						0.65	0.005	0.030	0.215	54.71
0.7	0.170	0.450	1.028	26.39						0.7	0.006	0.041	0.266	55.24
										0.75	0.007	0.056	0.323	55.37
										0.8	0.009	0.076	0.385	55.10
										0.85	0.011	0.101	0.451	54.40
										0.91	0.014	0.129	0.523	53.25

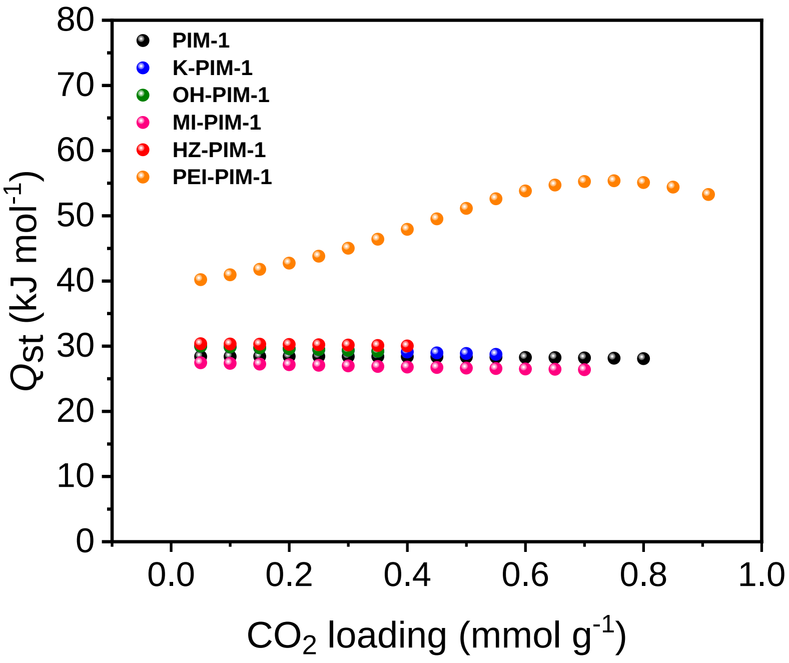

Supplementary Figure 38: Isosteric heat of adsorption (Q_{st}) values calculated from the adsorption isotherms at 273 K, 298 K and 323 K (Parameters derived from Logarithmic-scale plots for Dual-Site Langmuir-Freundlich (DSLF) model fitting for CO_2 adsorption isotherms at 273 K, 298 K, and 323 K by OriginPro, an alternative approach for calculation).

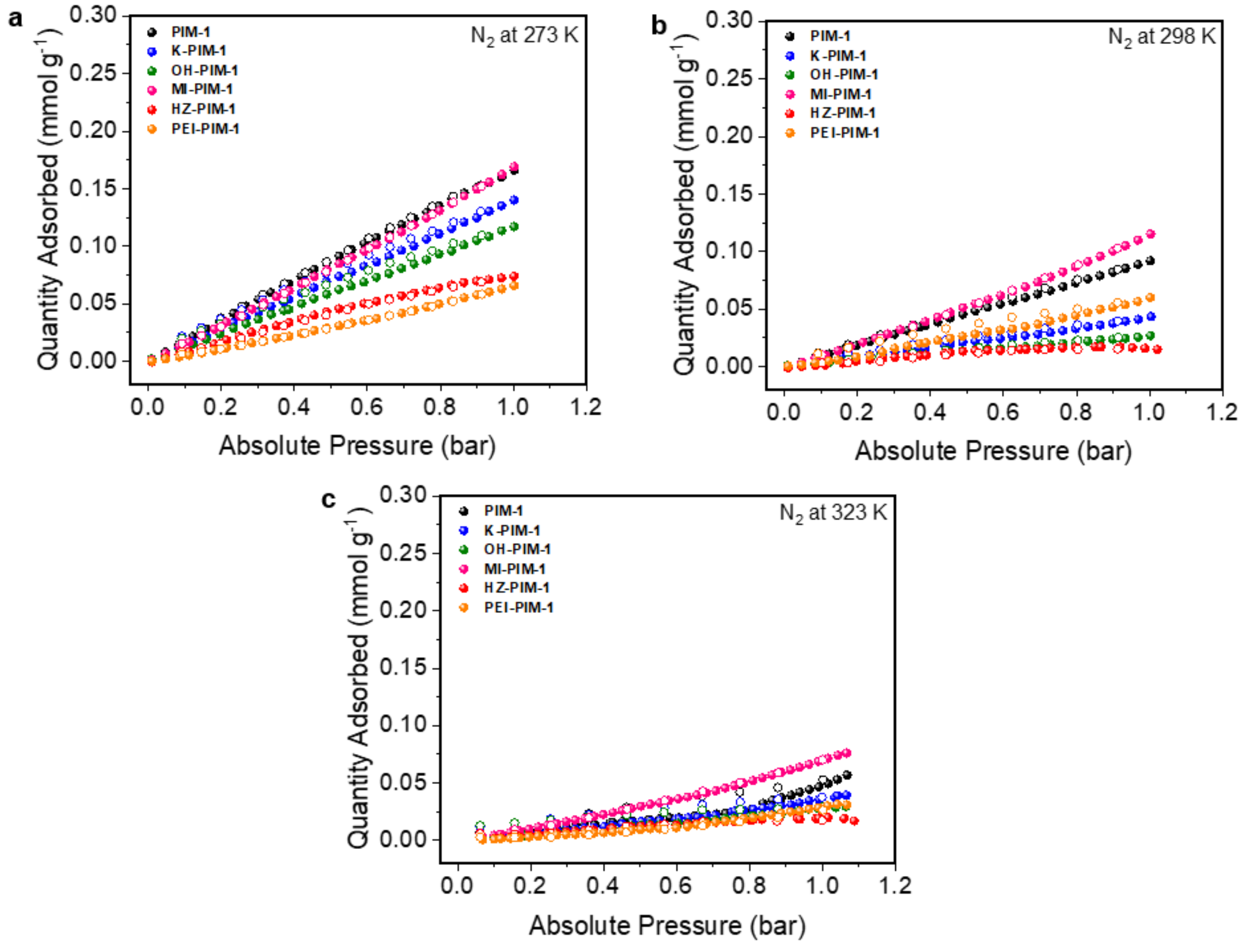

Supplementary Figure 39: Nitrogen adsorption-desorption isotherms of PIM-1 and derivatives at **a** 273 K, **b** 298 K, and **c** 323 K.

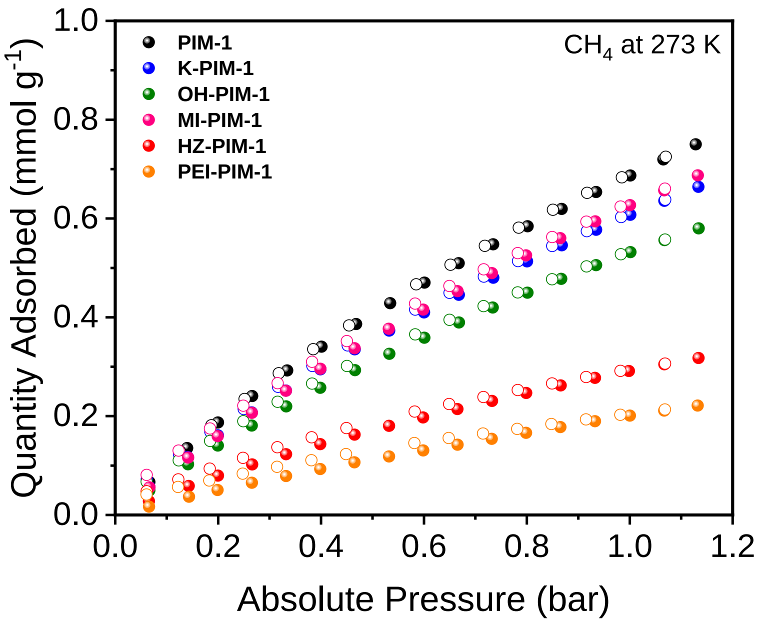

Supplementary Figure 40: Methane uptake isotherms of PIM-1 and derivatives at 273 K.

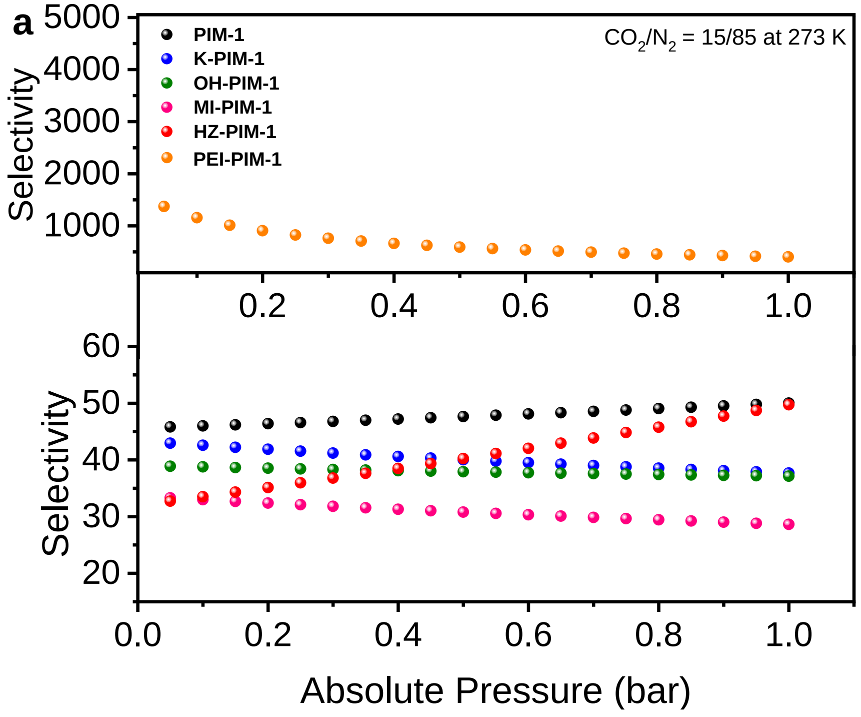

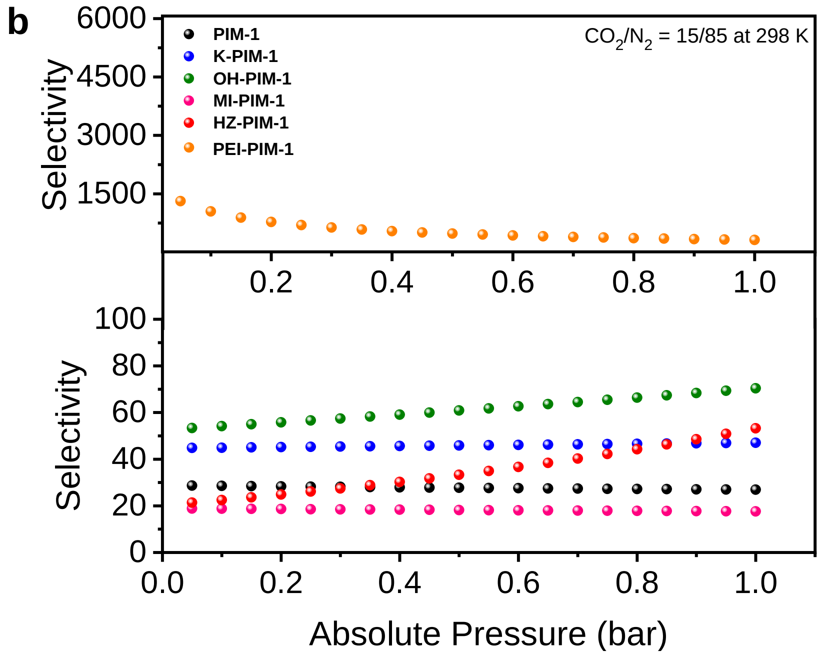

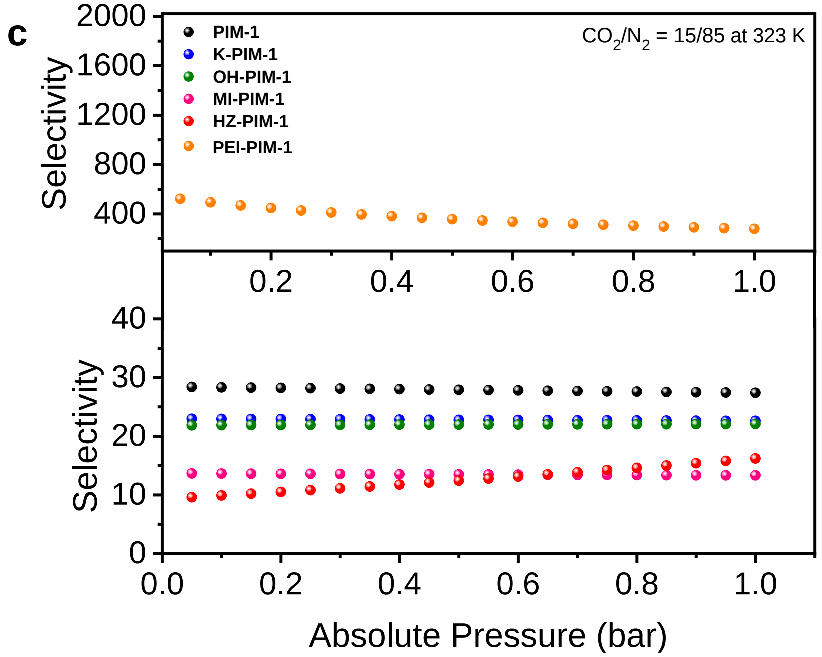

Supplementary Figure 41: Selectivity of CO_2 over N_2 of PIM-1 and derivatives. **a** at 273 K, **b** at 298 K, and **c** at 323 K predicted by the IAST method based on a CO_2 to N_2 molar ratio of 15 to 85.

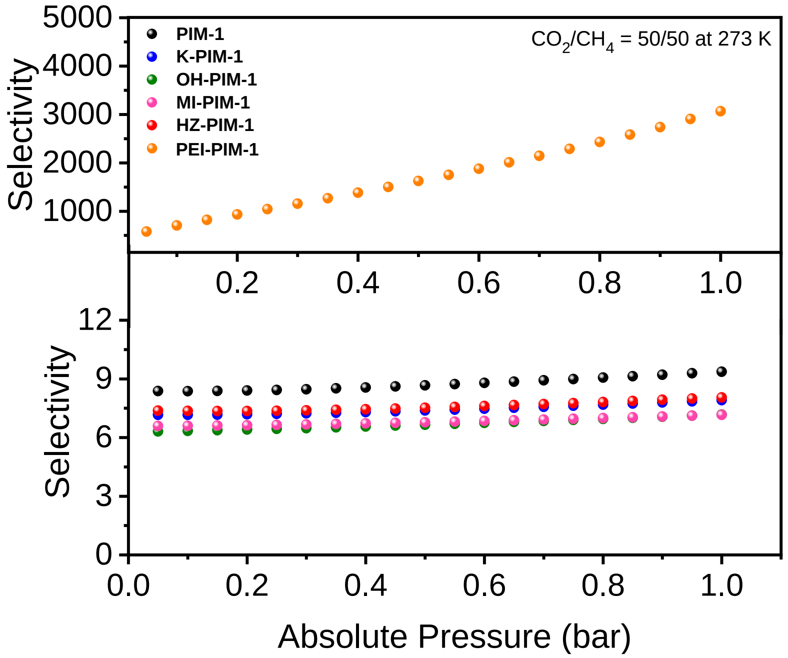

Supplementary Figure 42: Selectivity of CO_2 over CH_4 of PIM-1 and derivatives at 273 K predicted by IAST method based on a CO_2 to CH_4 molar ratio of 50 to 50.

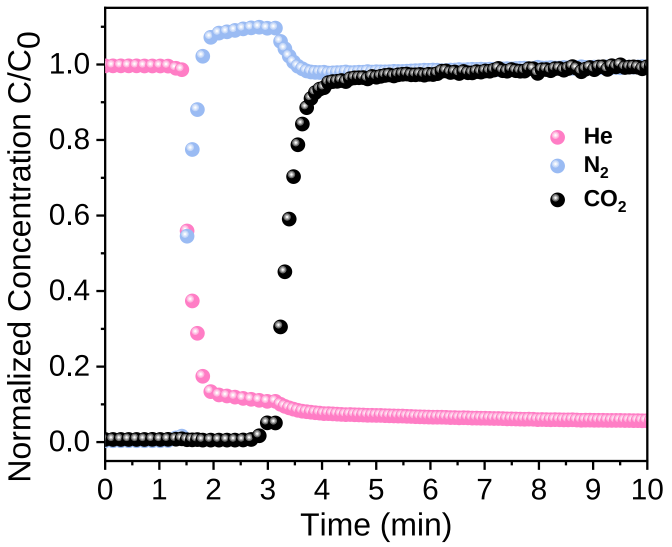

Supplementary Figure 43: Breakthrough curves of PIM-1 using $N_2/CO_2/He$ (80.75/14.25/5) gas mixtures at 1 bar and 298 K.

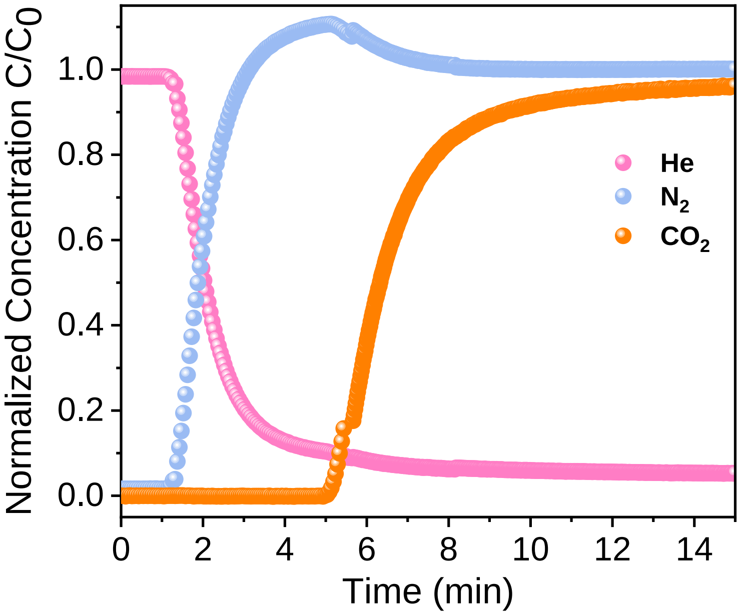

Supplementary Figure 44: Breakthrough curves of PEI-PIM-1 using $N_2/CO_2/He$ (80.75/14.25/5) gas mixtures at 1 bar and 298 K.

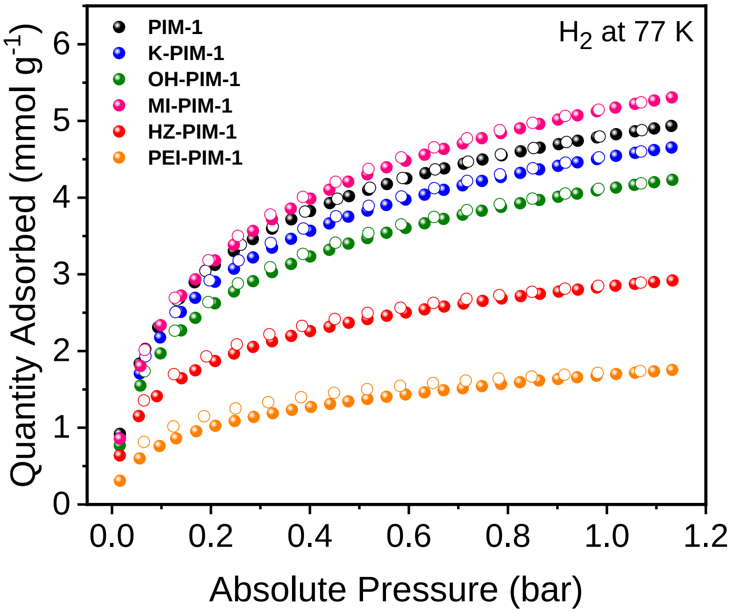

Supplementary Figure 45: Hydrogen uptake isotherms of PIM-1 and derivatives at 77 K.

Supplementary Table 15: Solubility test of PIM-1 derivatives.

Solvent	Relative Polarity	K-PIM-1	OH-PIM-1	MI-PIM-1	HZ-PIM-1*	PEI-PIM-1
Cyclohexane	0.006	+-	+-	-	-	-
Toluene	0.099	+-	+-	-	+-	-
Benzene	0.111	+-	-	-	-	-
1,4 dioxane	0.164	+-	+-	+-	+-	-
Tetrahydrofuran (THF)	0.207	+-	-	+-	+-	-
Chloroform	0.259	+-	+-	+-	+-	+-
Pyridine	0.302	+-	+-	-	+-	+-
Dichloromethane	0.309	+-	+-	+-	+-	-
Dimethylformamide (DMF)	0.386	+-	-	-	+-	-
Aniline	0.420	+-	+-	+-	+	+-
Dimethyl sulfoxide (DMSO)	0.444	+-	+-	+-	-	-
Isopropanol	0.546	-	-	+-	-	+-
Acetophenone	NA	+-	-	+-	+-	+-
N-Methyl-2-pyrrolidone (NMP)	NA	+-	+-	-	-	-
Nitrobenzene	NA	+-	+-	+-	+-	+-
Cyclopentanone	NA	+-	+-	+-	+-	-
Bromobenzene	NA	+-	+-	-	+-	+-
1,2 dichlorobenzene	NA	+-	+-	+-	+-	+-
Triethylenetetramine (TETA)	NA	+-	+-	+-	+	+-
m-cresol	NA	+-	+-	+-	+-	+-
Piperidine	NA	-	-	-	++	-
Quinoline	NA	+-	+-	+-	+	+-

- **Conditions**: Dissolve 10 mg of sample in 2 mL of solvent, sonicated at RT, and heating at 60 °C
- **Classification**: ++ soluble, + soluble after heating, +− partially soluble, − insoluble
- * The sample of HZ-PIM-1 was kept for over 1 month.

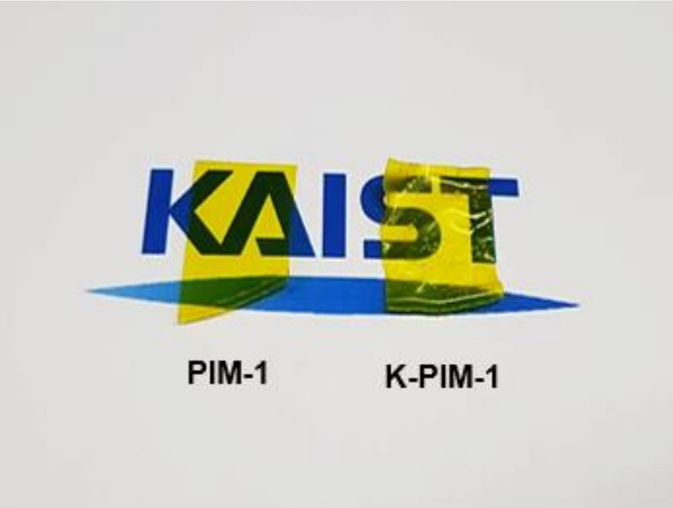

Supplementary Figure 46: Film formation of PIM and K-PIM-1.

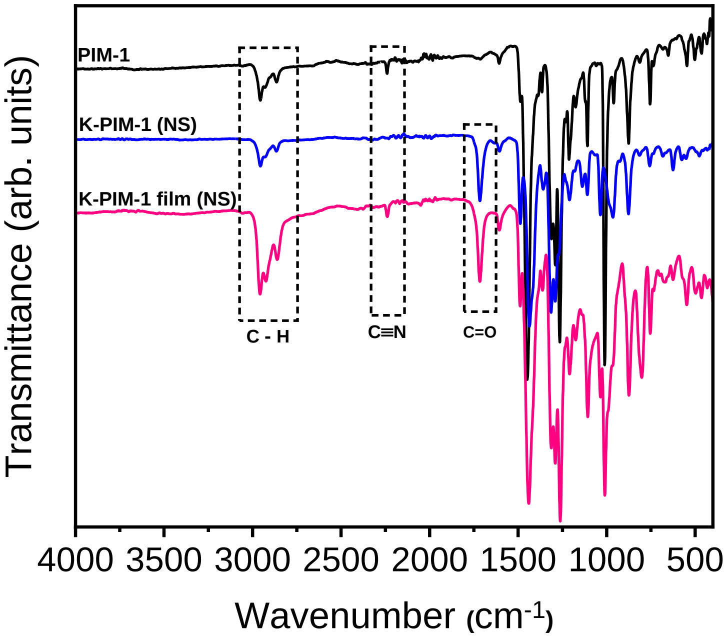

Supplementary Figure 47: FT-IR spectra of PIM-1, K-PIM-1, and K-PIM-1 film prepared by using the non-solvent method.

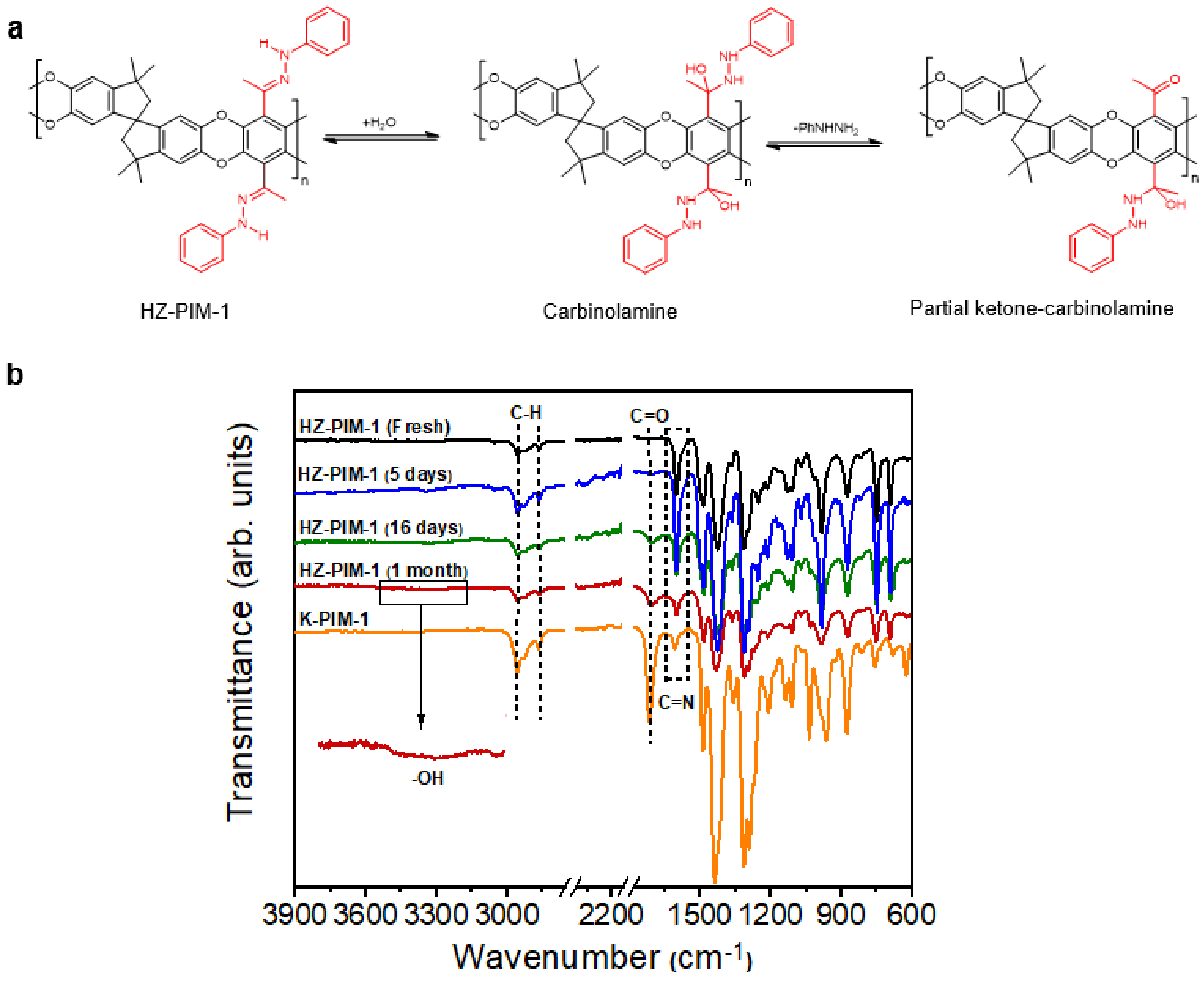

Supplementary Figure 48: a Mechanism and **b** FT-IR spectra revealed the reversible reaction of HZ-PIM-1.

Supplementary Table 16: Elemental (CHNO) analysis of HZ-PIM-1 at different storage times.

Storage time of HZ-PIM-1	Elemental analysis [%exp]			
	C	H	N	O
Partial ketone-carbinolamine [Theo.]	73.74	5.69	4.65	15.93
1 month	73.62±0.07	5.27±0.01	4.89±0.02	13.99±0.25
10 days	75.06±0.01	5.48±0.03	7.13±0.05	11.37±0.02
5 days	75.78±0.01	5.54±0.01	7.70±0.01	10.21±0.03
Fresh synthesis	75.06±0.10	5.58±0.00	7.74±0.01	9.95±0.02

7. Supplementary References

1. Patel, H.A. & Yavuz, C.T. Noninvasive functionalization of polymers of intrinsic microporosity for enhanced CO_2 capture. *Chem. Commun.* **48**, 9989-9991 (2012).

2. Budd, P.M. et al. Solution-processed, organophilic membrane derived from a polymer of intrinsic microporosity. *Adv. Mater.* **16**, 456-459 (2004).

3. Du, N.Y., Song, J.S., Robertson, G.P., Pinnau, I. & Guiver, M.D. Linear high molecular weight ladder polymer via fast polycondensation of 5,5',6,6'-tetrahydroxy-3,3,3',3'-tetramethylspirobisindane with 1,4-dicyanotetrafluorobenzene. *Macromol. Rapid Comm.* **29**, 783-788 (2008).

4. Yin, H.J. et al. First clear-cut experimental evidence of a glass transition in a polymer with intrinsic microporosity: PIM-1. *J. Phys. Chem. Lett.* **9**, 2003-2008 (2018).

5. Osterrieth, J.W.M. et al. How reproducible are surface areas calculated from the BET equation? *Adv. Mater.* **34**, 2201502-2201513 (2022).

6. Rouquerol, J., Llewellyn, P. & Rouquerol, F. Is the BET equation applicable to microporous adsorbents? *Stud. Surf. Sci. Catal.* **160**, 49-56 (2006).

7. Yanaranop, P., Santoso, B., Etzion, R. & Jin, J.Y. Facile conversion of nitrile to amide on polymers of intrinsic microporosity (PIM-1). *Polymer* **98**, 244-251 (2016).

8. Jeon, J.W. et al. Highly Carboxylate-Functionalized polymers of intrinsic microporosity for CO_2-selective polymer membranes. *Macromolecules* **50**, 8019-8027 (2017).

9. Mason, C.R. et al. Polymer of intrinsic microporosity incorporating thioamide functionality: preparation and gas transport properties. *Macromolecules* **44**, 6471-6479 (2011).

10. Du, N.Y. et al. Polymer nanosieve membranes for CO_2-capture applications. *Nat. Mater.* **10**, 372-375 (2011).

11. Xu, J.W. et al. Thiol-functionalized PIM-1 for removal and sensing for mercury (II). *J. Environ. Chem. Eng.* **8**, 104545 (2020).

12. Satilmis, B., Alnajrani, M.N. & Budd, P.M. Hydroxyalkylaminoalkylamide PIMs: selective adsorption by ethanolamine- and diethanolamine-modified PIM-1. *Macromolecules* **48**, 5663-5669 (2015).

13. Mason, C.R. et al. Enhancement of CO_2 affinity in a polymer of intrinsic microporosity by amine modification. *Macromolecules* **47**, 1021-1029 (2014).

14. Du, N.Y., Robertson, G.P., Dal-Cin, M.M., Scoles, L. & Guiver, M.D. Polymers of intrinsic microporosity (PIMs) substituted with methyl tetrazole. *Polymer* **53**, 4367-4372 (2012).

15. Wu, Q., Chen, S. & Liu, H. Effect of surface chemistry of polyethyleneimine-grafted polypropylene fiber on its CO_2 adsorption. *RSC Adv.* **4**, 27176-27183 (2014).

16. Lin, Y., Yan, Q., Kong, C. & Chen, L. Polyethyleneimine incorporated metal-organic frameworks adsorbent for highly selective CO_2 capture. *Sci. Rep.* **3**, 1859 (2013).

17. Liu, F.-Q. et al. Covalent grafting of polyethyleneimine on hydroxylated three-dimensional graphene for superior CO_2 capture. *J. Mater. Chem.* **3**, 12252-12258 (2015).

18. Zhu, J., Wu, L., Bu, Z., Jie, S. & Li, B.-G. Polyethylenimine-grafted HKUST-type MOF/polyHIPE porous composites (PEI@PGD-H) as highly efficient CO_2 adsorbents. *Ind. Eng. Chem. Res.* **58**, 4257-4266 (2019).

19. Liu, Z. et al. Moisture-resistant porous polymer from concentrated emulsion as low-cost and high-capacity sorbent for CO_2 capture. *RSC Adv.* **3**, 18849-18856 (2013).

20. Kolle, J.M. & Sayari, A. Covalently immobilized polyethylenimine for CO_2 adsorption. *Ind. Eng. Chem. Res.* **59**, 6944-6950 (2020).

21. Mane, S., Gao, Z.-Y., Li, Y.-X., Liu, X.-Q. & Sun, L.-B. Rational fabrication of polyethylenimine-linked microbeads for selective CO_2 capture. *Ind. Eng. Chem. Res.* **57**, 250-258 (2018).

22. Min, K., Choi, W., Kim, C. & Choi, M. Oxidation-stable amine-containing adsorbents for carbon dioxide capture. *Nat. Commun.* **9**, 726 (2018).

23. Zhou, Z. et al. Steam-stable covalently bonded polyethylenimine modified multiwall carbon nanotubes for carbon dioxide capture. *Energy & Fuels* **32**, 11701-11709 (2018).

24. Kassab, H. et al. Polyethylenimine covalently grafted on mesostructured porous silica for CO_2 capture. *RSC Adv.* **2**, 2508-2516 (2012).
